# Modulation of EEG theta by naturalistic social content is not altered in infants with family history of autism

**DOI:** 10.1038/s41598-022-24870-7

**Published:** 2022-12-01

**Authors:** Rianne Haartsen, Tony Charman, Greg Pasco, Mark H. Johnson, Emily J. H. Jones, The BASIS Team, Simon Baron-Cohen, Rachael Bedford, Anna Blasi, Patrick Bolton, Susie Chandler, Celeste Cheung, Kim Davies, Mayada Elsabbagh, Janice Fernandes, Issy Gammer, Holly Garwood, Teadora Gliga, Jeanne Guiraud, Kirstelle Hudry, Melanie Liew, Sarah Lloyd-Fox, Helen Maris, Louise O’Hara, Andrew Pickles, Helen Ribeiro, Erica Salomone, Leslie Tucker, Agnes Volein

**Affiliations:** 1 Centre for Brain and Cognitive Development, Birkbeck College, University of London, London, WC1E 7HX UK; 2 Department of Psychology, Institute of Psychiatry, Psychology and Neuroscience, King’s College London, De Crespigny Park, London, SE5 8AF UK; 3 South London and Maudsley NHS Foundation Trust, Bethlem Royal Hospital, Monks Orchard Road, Beckenham, Kent, BR3 3BX UK; 4 Department of Psychology, University of Cambridge, Cambridge, UK; 5 Department of Psychology, University of Bath, Bath, UK; 6 Department of Medical Physics and Biomedical Engineering, University College London, London, UK; 7 Department of Child and Adolescent Psychiatry, Institute of Psychiatry, Psychology and Neuroscience, King’s College London, London, UK; 8 Social Genetic and Developmental Psychiatry Centre, Institute of Psychiatry, Psychology and Neuroscience, King’s College London, London, UK; 9 NIHR Biomedical Research Centre in Mental Health at the Maudsley, London, UK; 10 Department of Psychiatry, McGill University, Montréal, Canada; 11 Psychology, School of Psychology, University of East Anglia, Norwich, UK; 12 Department of Psychology and Counselling, School of Psychology and Public Health, La Trobe University, Melbourne, VIC Australia; 13 Maudsley Biomedical Research Centre for Mental Health, London, UK; 14 Department of Psychology, University of Milan-Bicocca, Milan, Italy; 15 Present Address: ToddlerLab, Birkbeck, University of London, Malet Street, London, WC1E 7HX UK

**Keywords:** Psychology, Autism spectrum disorders

## Abstract

Theta oscillations (spectral power and connectivity) are sensitive to the social content of an experience in typically developing infants, providing a possible marker of early social brain development. Autism is a neurodevelopmental condition affecting early social behaviour, but links to underlying social brain function remain unclear. We explored whether modulations of theta spectral power and connectivity by naturalistic social content in infancy are related to family history for autism. Fourteen-month-old infants with (family history; FH; N = 75) and without (no family history; NFH; N = 26) a first-degree relative with autism watched social and non-social videos during EEG recording. We calculated theta (4–5 Hz) spectral power and connectivity modulations (social–non-social) and associated them with outcomes at 36 months. We replicated previous findings of increased theta power and connectivity during social compared to non-social videos. Theta modulations with social content were similar between groups, for both power and connectivity. Together, these findings suggest that neural responses to naturalistic social stimuli may not be strongly altered in 14-month-old infants with family history of autism.

## Introduction

The first year of postnatal human development is characterised by rapid developmental changes in cognition^1^. The second half of the first year is characterised by the development of more advanced social-cognitive skills such as responding to and initiating joint attention, language learning, and engaging in social games such as peek-a-boo. This is also the time-window in which behavioural signs of autism (including delays or the absence of these skills) first begin to emerge^2^. Autism is a neurodevelopmental disorder characterised by difficulty with social communication and interaction, patterns of restricted and repetitive behaviours, and sensory anomalies^3^. Although autism is typically diagnosed in childhood, the predominant expression of genes associated with autism in the pre- and postnatal brain^4,5^ suggests similar alterations in neural network function could be present from infancy. Substantial evidence indicates that the social and non-social symptoms of autism may emerge through distinct etiological mechanisms, and it may be fruitful to separately study their underpinnings^6,7^. In the mature adult brain, social interaction is underpinned by connected networks of specialized brain regions^8^; altered coordination of these brain networks has been hypothesized to contribute to social symptoms of autism^9^. Indeed, both hypo-activation within^10^, and decreases in connectivity between brain regions involved in social processing^11–13^ have been reported in autistic children and adults. Determining the degree to which social brain network changes precede the emergence of behavioural social symptoms is important to understanding their role in the causal aetiology of autism.

Alterations in brain development that precede the emergence of autistic behaviours can be identified within a prospective longitudinal infant sibling design^14,15^. In prospective longitudinal studies, infants with a family history of autism (typically an older sibling with the condition) are assessed across multiple visits in infancy and toddlerhood. About 20% of infants with a family history of autism themselves meet criteria for autism at age 3 years, and a further 30% have related developmental difficulties^16–19^. Infant neurodevelopmental measures can be evaluated in the context of family history, identifying endophenotypes that are present in infants with a genetic vulnerability regardless of diagnosis, and in the context of outcomes, identifying precursors of later symptoms. Such work has shown that infants with a later diagnosis of autism show relatively typical social skills in the first 6 to 12 months^2,20^ but show emerging social withdrawal and/or failure to acquire typical social milestones between 12 and 24 months^20–23^. These behavioural changes are preceded by differences in localized brain responses to social stimuli. For example, 4- to 6-month-old infants with a family history of autism showed reduced responses to vocal (non-speech sounds) versus non-vocal (environmental sounds) auditory stimuli in the mid-posterior superior temporal sulcus and to social dynamic videos versus static images of transport types (like cars or helicopters) in the posterior temporal cortex^24^. These responses were also related to later diagnostic outcome at 36 months in a follow-up study: infants with a later diagnosis of autism showed reduced activation in the inferior frontal gyrus and posterior temporal cortex to the social dynamic videos versus the static non-social images and reduced activation in the left lateral temporal regions during vocal versus non-vocal auditory stimuli when compared to infants without a later diagnosis^25^. Activation for both visual and auditory stimuli at infancy was related to severity of autism symptoms at toddlerhood. Further, infants with family history and later autism also show altered event-related neural responses to static faces with direct and averted gaze at 6–9 months^26^ and 10 months^27^ of age and in other cohorts^28,29^. Thus, both temporal and spatial brain responses to social stimuli may be altered prior to the emergence of overt social communication difficulties^30,31^.

Less is known about the emergence of specific networks of social brain regions. Recent work with infant sibling designs has shown associations between alterations in infant brain networks and autistic symptoms within the non-social domain. Infants with and without a family history of autism watched naturalistic videos at 14 months of age. Infants who met criteria for autism at 36 months of age displayed elevated connectivity in fronto-central regions in the alpha band (7–8 Hz). This increased infant alpha connectivity was associated with increased severity of restrictive and repetitive behaviours at later age^32^. The association between infant alpha connectivity and later restricted and repetitive behaviours in infants was replicated in an independent cohort^33^. This suggests that alpha connectivity may be associated with symptomatology in the non-social domain. However, network connectivity has not yet been related to autistic-related differences in social functioning in infancy.

A recent line of work suggest theta power and connectivity may be modulated by the social content of naturalistic scenes in infancy. Jones et al.^34^ examined theta and alpha power while infants watched naturalistic dynamic videos. In the ‘social’ dynamic videos, women sang nursery rhymes directed to the infants while making accompanying gestures—such as ‘peek-a-boo’, ‘pat-a-cake’, and ‘incy-wincy spider’. In the ‘non-social’ dynamic videos, colourful toys were moving, such as balls falling down a ball drop and a spinning top, with the naturalistic congruent clanging sounds made by the toys. The ‘non-social’ dynamic videos were used as a control to compare to the ‘social’ dynamic videos to control for facial and biological motion while being more complex than static images of types of transport as used in previous infant imaging studies^24,25,35^. In addition, the dynamic videos with congruent visual and auditory stimulation provide a more naturalistic context with higher ecological validity compared to static images. For consistency with the previous studies using this paradigm, we will use the terms ‘social’ and ‘non-social’ videos to refer to the dynamic videos with the signing women and the dynamic videos with spinning toys, respectively^24,25,32,34,36–38^.

Jones and colleagues found that at 12 months of age, theta power (3 to 6 Hz) was increased during social compared to non-social videos in occipital and frontal regions but not temporal and parietal regions. Alpha power (6 to 9 Hz) showed no differences between conditions at this age. Further, theta power was increased when infants looked at the experimenter’s face compared to the toys in the experiment’s hand during the live condition while the experimenter was singing nursery rhymes throughout. Another study in neurotypical infants used similar social (Dutch versions) and non-social dynamic videos in a longitudinal study during the 5- and 10-month visits. The findings revealed increasing differentiation with age in theta EEG connectivity (3 < 6 Hz) between conditions: theta connectivity increased across the whole brain during social compared to non-social videos. This differentiation started to emerge from 6 months of age and showed a medium effect from 9 months of age. EEG connectivity within the alpha range (6 < 9 Hz) did not vary between the dynamic videos^39^. Finally, infant changes in theta oscillations have been previously related to later individual differences in socio-communicative development. Individual differences in left frontal theta power and EEG connectivity (4–6 Hz) in typically developing 14-month-old infants while viewing colourful balls in a rotating bingo wheel predict later joint attention skills and vocabulary at toddlerhood^40,41^. In young children with autism, increased theta power (5–7 Hz) for static faces compared to static objects was linked to individual differences in social symptoms^42^. Thus, examining EEG oscillatory theta modulations during social and non-social naturalistic videos may be a fruitful way to investigate early social brain development in infants with a family history of autism.

In the current study, we examined differences in theta EEG power and EEG connectivity between naturalistic social and non-social videos in 14-month-old infants with and without a family history of autism. We chose for these videos as the combined visual and auditory dynamic stimuli are more naturalistic than static pictures of faces or houses, or vocal or non-vocal sounds used in previous studies. We analysed data from 2 previous cohorts in our prospective longitudinal infant sibling study. We decided to focus on the 14-month time point as previous studies suggest differences between videos in theta power and connectivity start to emerge around the first birthday in typically developing infants^34,39^, and behaviorally-measured social difficulties start to emerge between the first and second birthday in infants with later autism^20–23^. Further, we focus on family history to examine endophenotypes of autism that may reflect subclinical features and different behavioural outcomes from multi-final pathways^43,44^. Of note, we have previously shown that within these 2 cohorts, fronto-central EEG connectivity in the **alpha** band (7–8 Hz) replicably related to later restricted and repetitive behaviours across collapsed social and non-social videos^32,33^; theta power and EEG connectivity (4–5 Hz) across collapsed conditions in the 14-month-olds did not differ between the infants with and without a later diagnosis of autism^32^, but the modulation of theta power and connectivity by condition was not examined due to small sample sizes. Here, we collapse the data from 2 cohorts to examine condition modulations for theta power and connectivity (4–5 Hz). In addition, we assessed severity of autistic symptoms in the social domain at 36 months of age. We decided to use the same sample of infants for all analyses to be able to compare power and connectivity findings in the same sample. This would also allow us to keep the results concise rather than using different samples for power and connectivity analyses which would further complicate the study.

If early social brain network development is altered in infants with a family history of autism, we expected differences in how theta power and connectivity are modulated by social content between the NFH and FH groups. Based on previous work with neurotypical infants we expected to find increases in frontal and occipital theta power and whole brain theta connectivity during social versus non-social videos^34,39^. We hypothesized that theta power and connectivity would differ more between social and non-social videos in the NFH than FH group^34^. In addition, if early social brain network alterations precede behavioural social symptoms of autism, we expect to find a correlation between variability in early neural modulations with social content and variability in clinical measures of social symptoms but not non-social symptoms at later age.

## Results

### Participants

**Figure 1:**
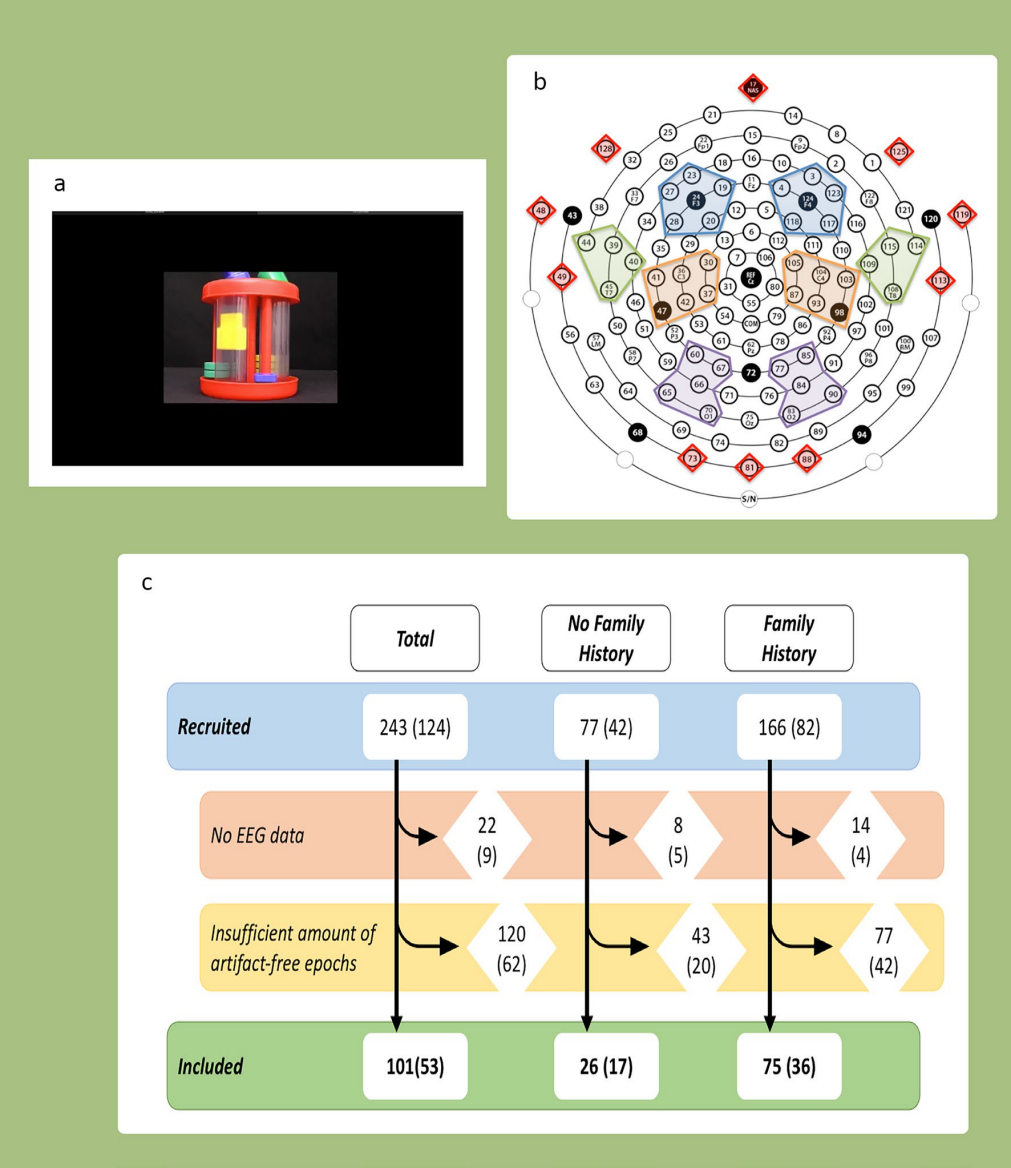
Paradigm and attrition rates. (**a**) Screenshot of one of the toy dynamic videos. (**b**) Top view of the layout of the EEG high-density net. Electrodes on the outer rim were excluded since these were bad during the recording (red diamonds). Spectral theta power was calculated for frontal (blue), parietal (orange), temporal (green), and occipital (purple) regions (after^34^). (**c**) Attrition rates for the samples included in the analyses.

Our final sample of included participants consisted of 26 (17 females) NFH infants and 75 (36 female) FH infants, after exclusion of infants who did not have EEG data available for the task or insufficient amounts of artifact free epochs (see Fig. 1 and “Methods” section). Table 1 displays the participant demographics for the included sample. The NFH group showed higher developmental levels at both the 14- and 36-month-old visit (*p*s ≤ .017) as measured by the Mullen Scales of Early Learning, Early Learning Composite Standard Score (MSEL ELC), while the ages of assessments were similar between groups at both visits. The NFH group displayed better social skills than the FH group at toddlerhood as reflected by higher scores on both domains of the Vineland Adaptive Behaviour Scale (VABS)-II: Communication and Socialisation (*p*s ≤ .002). The FH group showed more severe autistic traits on the social and non-social domain than the NFH group, as indicated by the higher scores on the Social Responsiveness Scale (SRS)-2 for the Social Communication and Interaction (SCI), and the Restricted and Repetitive Behaviours (RRB) domain (*p*s < .0001).

**Table 1:** Demographics of the sample for comparisons between the social and non-social condition.

	NFH	FH	*p* value
Number of participants (female)	26 (17)	75 (36)	.126
**At 14-month-old visit**			
Age in days	462 (369–543)	458 (361–567)	.305
MSEL ELC	104 (15)	96 (14)	.017*
**At 36-month-old visit**			
Age in months	38 (37–41)	38 (34–42)	.836
MSEL ELC	122 (103–143)	106 (43–173)	.004**
VABS-II Communication	110 (73–145)	100 (63–135)	.002**
VABS-II Socialization	106 (96–116)	97 (64–128)	< .0001***
SRS-2 SCI	40 (29–53)	46 (26–68)	< .0001***
SRS-2 RRB	40 (37–45)	44 (29–65)	< .0001***

Medians with the lower and upper quartile boundaries in parentheses are given for all measures at the 14 and 36-month-old visits, except for MSEL ELC scores at the 14-month-old visit for which the mean and standard deviation in parentheses are given. Significant comparisons (at the α level = .05) are printed in bold.

MSEL ELC—Mullen Scale for Early Learning Early Learning Composite Standard Score; SRS-2 RRB—Social Responsiveness Scale-2, T-score for the Restricted and Repetitive Behaviours domain; SRS-2 SCI—Social Responsiveness Scale-2, T-score for the Social Communication and Interaction domain; VABS-II Communication—Vineland Adaptive Behaviour Scale-II, Communication domain standard score; and VABS-II Socialization—Vineland Adaptive Behaviour Scale-II, Socialization domain standard score.

### Theta power modulations with naturalistic videos

**Figure 2:**
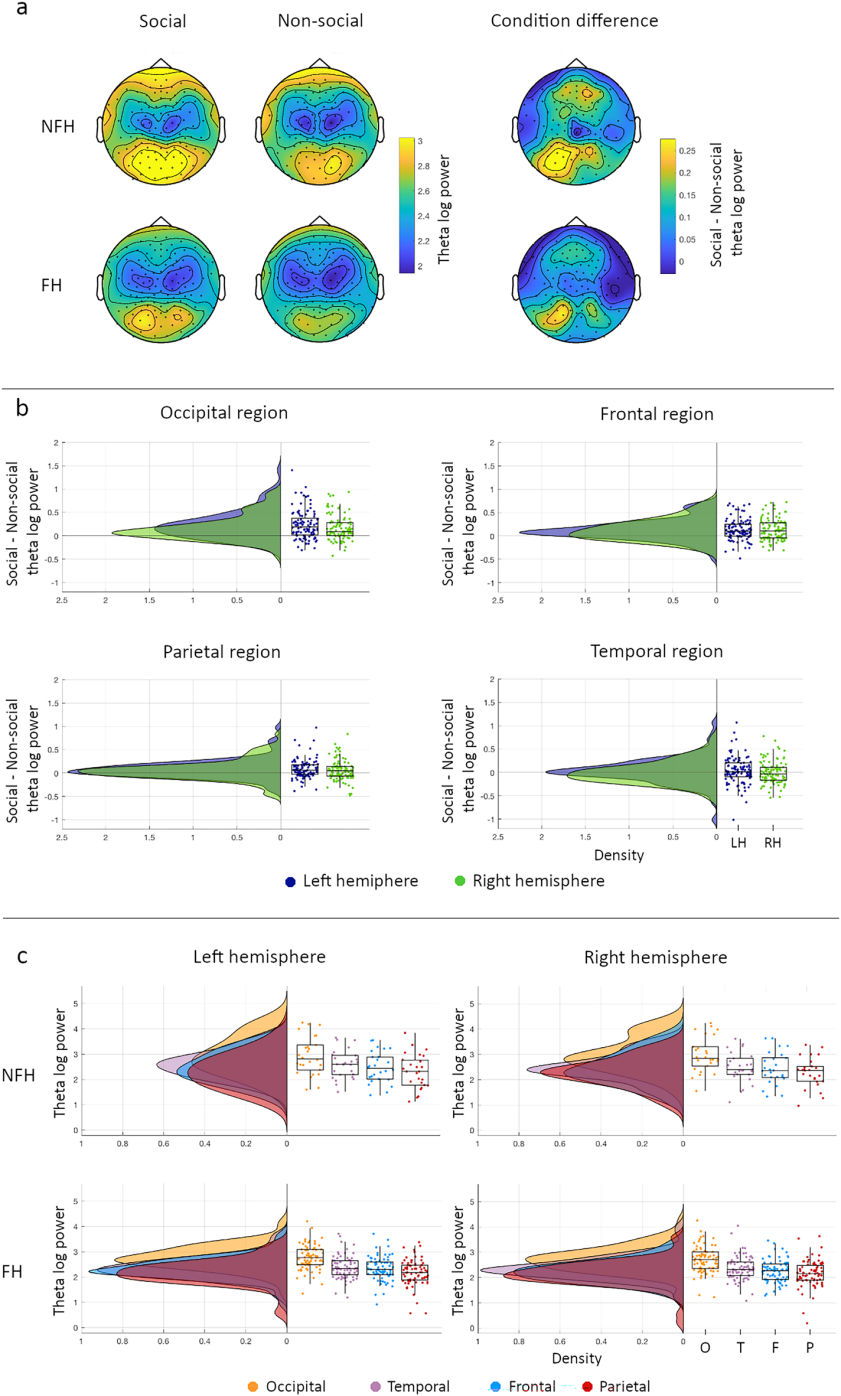
Theta power modulations by social context. (**a**) Topoplots for the social and non-social condition (left and middle) and the difference (social–non-social) between conditions (right) for the NFH (top) and FH (bottom) group. (**b**) Density and boxplots for condition differences in power for the left (LH in navy) and right (RH in green) hemisphere in for each region (occipital in top left, frontal in top right, parietal in bottom left, and temporal in bottom right plot). Each dot represents 1 infant. Positive values indicate higher power for social than the non-social condition. (**c**) Density and boxplots for power within each region (occipital (O) in yellow, Temporal (T) in purple, Frontal (F) in blue, and Parietal (P) in red) within the left hemisphere (left) and the right hemisphere (right) for the NFH (top) and the FH (bottom) group. Each dot represents 1 infant.

Topoplots for the family history groups for each condition and the condition differences are presented in Fig. 2a. We conducted a 2 × 4 × 2 × 2 mixed GLM using Condition (Social, Non-social), Region (Frontal, Temporal, Parietal, Occipital), and Hemisphere (Left, Right) as within-subject factor as in^34^, and Group (NFH, FH) as between-group factor. Theta power was higher for the social than the non-social condition (*p* < .0001, *η*_*p*_^2^ = .195). Theta power varied with region (*p* < .0001, *η*_*p*_^2^ = .646), showing highest values for occipital, then temporal and frontal regions, and lowest values for parietal regions. Theta power was higher in the left than right hemisphere (*p* = .014, *η*_*p*_^2^ = .059). There was no overall difference in theta power between family history groups (*p* = .133, *η*_*p*_^2^ = .023).

There were significant interactions between Condition and Region (*p* < .0001, *η*_*p*_^2^ = .288); Condition and Hemisphere (*p* = .002, *η*_*p*_^2^ = .091); Condition, Region, and Hemisphere (*p* = .037, *η*_*p*_^2^ = .030); and Region, Hemisphere and Group (*p* = .026, *η*_*p*_^2^ = .031). Briefly, condition effects were largest over occipital regions (*p* < .0001, *η*_*p*_^2^ = .346), then frontal (*p* < .0001, *η*_*p*_^2^ = .275), then parietal (social > non-social, *p* < .0001, *η*_*p*_^2^ = .119), and not significant over temporal regions (*p* = .454, *η*_*p*_^2^ = .006, Fig. 2b). Greater condition differences over left versus right hemisphere were seen over occipital regions (*p* < .0001, *η*_*p*_^2^ = .176) but not parietal (*p* = .054, *η*_*p*_^2^ = .037), temporal (*p* = .071, *η*_*p*_^2^ = .032) or frontal regions (*p* = .917, *η*_*p*_^2^ = 0). Follow-up tests of interactions between Region, Hemisphere and Group did not reveal clear patterns but suggested different topographies of overall theta power by group with greater lateralisation in the FH (*p* = .003, *η*_*p*_^2^ = .112) than the NFH group (*p* = .320, *η*_*p*_^2^ = .040, Fig. 2c, also see SI1 section. 1.1 for more details).

To summarise the pattern of results, we found increased theta power during social videos compared to non-social videos with stronger increases in occipital than frontal than parietal regions. Groups with varying family history of autism may present differences in overall topographies, but follow-up tests did not show a clear pattern. These results were not influenced by data quantity, ratio of data quantity across conditions, age, sex, or developmental levels of the infants (see SI1 section 2.1).

### Theta connectivity modulations with naturalistic videos

**Figure 3:**
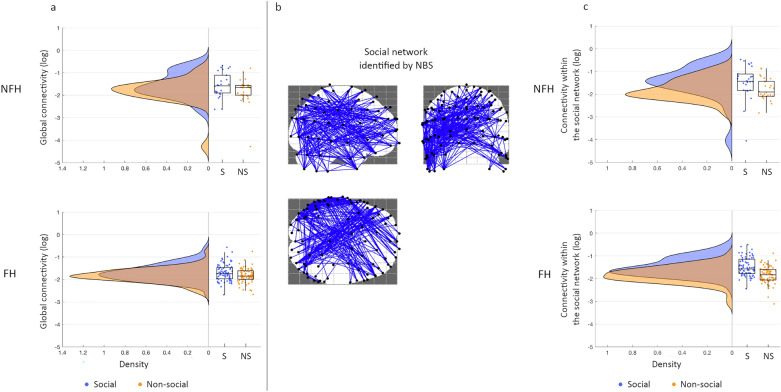
Theta connectivity modulations by social context. (**a**) Density and boxplots for global connectivity for the social (blue) and non-social (orange) condition in the NFH group (top) and FH group (bottom). Each dot represents 1 infant. (**b**) Network of edges and nodes showing elevated connectivity during the social versus non-social condition. (**c**) Density and boxplots for connectivity within the social network for the social (blue) and non-social (orange) condition in the NFH group (top) and FH group (bottom). Each dot represents 1 infant.

A 2 × 2 mixed ANOVA (Condition x Group) with global connectivity as dependent measure (debiased weighted phase lag index (dbWPLI) values averaged across all possible channel pairs) revealed that global connectivity was elevated during the social condition compared to the non-social condition (*p* < .0001, *η*_*p*_^2^ = .126, see Fig. 3a). Global connectivity did not vary between groups with and without family history of autism (*p* = .380, *η*_*p*_^2^ = .008), nor did condition modulations vary by group (*p* = .216, *η*_*p*_^2^ = .015).

Next, we examined whether any channel pairs would be consistently modulated by condition in the whole sample using Network Based Statistics (NBS). We found a network with 262 edges (channel-pairs) and 95 nodes (channels; *p* < .001) that showed elevated connectivity during social condition compared to non-social condition (Fig. 3b). This ‘social’ network showed a topography with strong connectivity between frontal, left temporal, and left occipital channels (the 25% of the channel-pairs showing the strongest condition difference). In follow-up analyses, we calculated the social network connectivity (i.e., average connectivity within the network showing elevated connectivity during social videos for each individual) and compared this modulation between family history groups using a 2 × 2 mixed ANOVA (Condition x Group). Connectivity within the social network was elevated during the social condition versus the non-social condition (*p* = .273, *η*_*p*_^2^ < .0001, Fig. 3c), as expected. The family history groups did not show any differences in connectivity within the social network (*p* = .681, *η*_*p*_^2^ = .002), or in modulation with condition (*p* = .863, *η*_*p*_^2^ = 0).

Finally, we tested whether any channel pairs showed an interaction effect between family history group and condition using NBS. The results revealed no social networks that varied with family history.

This suggests that theta connectivity varies with social context. Modulations within the whole brain and connections showing consistent modulations with social content are similar for groups of infants with varying family history of autism. Technical factors such as data quantity and ratio of data quantity across conditions, or demographic factors such as age, sex, or developmental levels of the infants did not influence the main pattern of findings (see SI1 section 1.3).

### Associations between theta modulations and later behaviours

In addition to categorical group analyses, we were interested in associations between the neural modulations by condition and severity of later behaviours characteristic of autism. We calculated the modulation between conditions for the average connectivity within the social network (see above), and for theta power averaged across all regions of interest. We then correlated these neural measures with behavioural measures of social symptoms (VABS-II Communication and Socialization domain, and SRS-2 SCI), and non-social symptoms (MSEL ELC, and SRS-2 RRB).

Table 2 displays the results for these correlations. None of the associations tested reached significance.

**Table 2:** Spearman’s correlations between modulations of connectivity and power with later behaviours at the 36-month-old visit.

	*N*	Connectivity modulation within the NBS network		Overall power modulation	
		Rho	*p* value	Rho	*p* value
**Social domain**					
VABS-II Communication	97	− .03	.806	− .10	.331
VABS-II Socialization	97	.01	.892	.04	.725
SRS-2 SCI	93	.03	.813	− .10	.367
**Non-social domain**					
MSEL ELC	97	.04	.729	.10	.347
SRS-2 RRB	93	− .06	.567	− .11	.299

Significant comparisons (at the α level = .05) are printed in bold.

MSEL ELC—Mullen Scale for Early Learning Early Learning Composite Standard Score; SRS-2 RRB—Social Responsiveness Scale-2, T-score for the Restricted and Repetitive Behaviours domain; SRS-2 SCI—Social Responsiveness Scale-2, T-score for the Social Communication and Interaction domain; VABS-II Communication—Vineland Adaptive Behaviour Scale-II, Communication domain standard score; and VABS-II Socialization—Vineland Adaptive Behaviour Scale-II, Socialization domain standard score.

## Discussion

We investigated theta power and connectivity modulations during naturalistic social and non-social videos in infants with and without a family history of autism, and whether or not variation in neural modulations by social content were associated with social development outcomes in toddlerhood. Both theta power and connectivity were elevated in 14-month-old infants when they viewed videos of women singing nursery rhymes (social condition) versus when they viewed videos of toys moving (non-social condition). Power modulations with condition were similar between family history groups. Connectivity was elevated across the whole head and within a specific social network during the social videos compared to the non-social videos. Again, these modulations did not differ between family history groups. The current findings extend previous findings of power and connectivity modulations with social naturalistic content from neurotypical infants to those infants with a family history of autism.

As predicted^34^, both theta power and connectivity were increased during dynamic social stimulation compared to non-social stimulation. The results for theta power in the NFH group are partially consistent with previous findings from Jones and colleagues in 12-month-old neurotypical infants^34^. As in the 12-month-olds in this previous theta power study, we observed differential brain responses between social and non-social videos in the frontal and occipital regions, but not the temporal region. We did find condition differences in the parietal region at 14 months in the current study while this effect was not present at 12 months. It is possible that this additional region is an extension in the topography of the socially sensitive theta response with age. There was no effect of video condition in any of the regions in neurotypical 6-month-olds whereas there were in frontal and occipital regions in the 12-month-olds. According to the interactive specialisation hypothesis, neural responses become more specific to certain stimuli and this selectivity becomes more widespread with age^31,45^. The hypothesis further suggests that connectivity would be increased and more widespread in older infants. Previous findings have shown that connectivity modulations with videos also increase with age between 5 and 10 months^39^. The current findings demonstrate that infants at 14 months also show this modulation. Further studies using longitudinal data are needed to examine the development of specialisation of neural responses to social stimuli in neurotypical infants.

Modulations in theta oscillations have been observed in a range of studies with varying interpretations for cognitive functions. For example, theta oscillations have been associated with active learning and the anticipation and processing of reward^46–48^. Theta power is increased when infants are anticipating or expecting to learn from a social partner^49–51^. Another interpretation is that the theta oscillations reflect attentional control or the allocation of attention ^48,52^. Increases in theta oscillations in frontal and temporal areas during listening to stories^53^, exploring objects^53^, or solo play^54^ are related to attentional engagement to direct activation in different brain networks. Theta power increases in the orbitofrontal lobe during sustained attention versus stimulus orienting and attention termination (with attention states defined by heart-rate measures ^55^). Theta oscillations from frontal areas may implement top-down control over sensory processing areas, such as the visual regions, and integrate multi-sensory information through long-range neuronal communication^52^. The prefrontal cortex has been suggested to play a role in modifying the neural processing in sensory areas^31,56^. Finally, modulations in theta oscillations have been observed during language processing and may relate to neural tracking where neural oscillations synchronise in phase with the perceived speech, or in this paradigm, the nursery rhymes^57,58^. Modulations of neural amplitudes across the whole brain during listening to nursery rhymes are particularly observed in the delta band with a peak at ~ 2 Hz from 4 months, and in the theta band at ~ 4 Hz with the strongest response at 11 months of age compared to the younger ages tested^59,60^. In previous work, 10 and 14-month-old infants show neural tracking during sung nursery rhymes (using a similar paradigm as Jones et al.^34^). Thus, theta oscillations are sensitive to a range of different cognitive processes.

Given this broad literature, we cannot determine the exact features of the complex dynamic videos in the current study that explain the observed theta modulations in power and connectivity. It is possible that more active learning and social reward processing occurs during the social compared to the non-social videos. Alternatively, the social videos may have elicited increased attentional control for integrated processing of social stimuli relative to the non-social videos. Increased attention to the auditory speech stimuli in the social videos compared to mechanical clanging sounds in the non-social videos may also account for the observed theta differences. Neural tracking of the speech envelope may further explain the results. Although we did not observe a strong peak at ~ 2 Hz in our graphs, it is possible that the differences in theta synchronisation are a result of the neural synchronisation to the speech envelope of the nursery rhymes. (Note, differences in theta power between conditions during infancy were not related to concurrent or later language skills. Also, see SI1 1.4) Finally, a combination of these factors may have played a role in the theta modulations with social context found in the current study. Large-scale increased connectivity or consistency of phase-coupling may reflect increased communication between brain regions involved in learning and reward processing, attentional control, and in the visual and auditory processing of the social videos. The increased communication may furthermore facilitate integration between top-down and bottom-up processing^61–64^. Our selection of the social and non-social dynamic videos was based on their high ecological validity; but the disadvantage of this approach is it does not allow us to dissociate between the different possible explanations. Further research is needed to disentangle the possible effect on theta oscillations from the sensory input (visual stimuli and auditory stimuli) and higher-order cognitive functions (learning, attention, information integration).

Our findings further extend previous findings in neurotypical infants by examining theta modulations with social content in infants with and without a family history of autism. In contrast to our predictions, we did not find differences in theta modulations between the family history groups. This finding suggests that theta oscillatory responses to social naturalistic stimuli may be similar across infants with varying family history of autism at 14 months of age. Our supplementary analyses comparing theta modulations between group with varying family history and outcomes (NFH vs FH- typical development/ TD vs FH- no typical development/noTD) revealed similar patterns (see SI2). Theta power modulations displayed subtle differences in topographies between the outcome groups, and further work is needed to examine topographic variation using more topographically sensitivity measures, such as topographic analyses of variance (TANOVA) and microstate analyses^65–70^. However, there were no differences in connectivity modulation within the social network between infants with and without a family history of autism. This is consistent with some previous studies. For example, NFH and FH infants displayed similar speech-brain coherence while viewing social videos at 14 months, and this neural tracking was not related to later autism symptom severity at 36 months of age^71^. Later in development, children with a diagnosis of autism may show differences in social brain networks: in one study, autistic 3-year-olds displayed stronger driving theta oscillations from regions within the social brain compared to their non-autistic peers. The strength of these oscillations was related to concurrent socialisation skills (VABS-II scores for the Socialisation domain)^72^. Previous findings in connectivity in infants with and without family history or later diagnosis of autism are mixed. Theta phase coherence while listening to an auditory statistical learning paradigm was further lower in 3-month-old infants with high scores on an autism assessment during their 18-month-visit compared to 3-month-olds with low scores at later age^73^. Linear phase coherence in the gamma frequency range while listening to speech sounds showed no differences between family history groups at 6 months but was lower in the FH than the NFH group at 12 months. Further, coherence was lower in infants with a diagnosis of autism at a later age compared to infants without a later diagnosis^74^. Future research is needed to examine if sensitivity to social stimuli changes across development in infants with varying developmental outcomes.

Our findings on theta power suggested infants without a family history of autism displayed a different power topography overall compared to infants with a family history of autism.

In contrast to our predictions, we did not observe any robust associations between infant theta power or connectivity modulations and behavioural measures at toddlerhood across the sample. Associations between neural modulations and dimensional outcomes may vary with developmental stage and frequency band of interest. Previous studies have associated frontal theta power during the first year of life to language skills during toddlerhood^75–77^; our findings may differ since we measured EEG modulations shortly after the first year (at 14 months of age). Further, in a previous report with an overlapping sample, we had also observed a network of fronto-central connections within the alpha range that specifically and reliably related to later restricted and repetitive behaviours in the FH-Autism group, suggesting that connectivity within different frequency ranges may have a degree of phenotypic specificity^32,33^. Concurrent EEG and fMRI or fNIRS data could indicate whether our identified alpha and theta networks reflect the activity of different brain regions, or different frequencies of communication within the same network. Future research could focus on examining developmental changes in theta power and connectivity modulations by social content over time.

We note use of sensor rather than source space connectivity analyses; and the underestimation of short-range connectivity by the dbWPLI as being limitations of this study^32,33,78,79^. Source-localised data would allow for graph theory analyses which may inform on the organisational structure of underlying brain networks. Further, it is challenging to disentangle the underlying function of theta power and what cognitive function it reflects. It is possible that a combination of anticipation of reward processing, active learning, attentional control, and language processing elicits increases in theta oscillations. The dynamic social and non-videos presented here are suitable for paediatric and neurodevelopmental research, but their ecological validity comes at the cost of experimental control of specific visual or auditory features, making it difficult to pinpoint the exact features that modulate theta power. To ensure sufficient data quantity, videos were repeated; we are unable to examine repetition effects because of limited segment numbers. Toy and hand conditions were collapsed because they were visually substantively similar (see “Methods” section), data quantity increases, and because preliminary analysis suggested no meaningful differences, but this meant the non-social stimulus did contain a small social element which may have slightly reduced effect sizes.

To conclude, we contrasted the strength and connectedness of brain activity during social and non-social experiences in relation to both categorical and dimensional developmental outcomes. Our findings replicate previous results and suggest that brain activity strength and connectivity in the theta frequency range are sensitive to social context. These modulations with social context are similar in infants with varying family history of autism. Together, the results show that theta social brain networks appear relatively robust at 14 months in infants with a family history of autism compared to infants without a family history of autism. Future studies using multimodal imaging techniques and taking a systems neuroscience approach may further elucidate underlying mechanisms that may help to enrich intervention for infants with varying familial history of autism.

## Methods

### Participants

Current analyses are based on data collected across 2 cohorts as part of the British Autism Study in Infant Siblings (BASIS) study (www.basisnetwork.org), a longitudinal prospective study of infants with and without a family history of autism with repeated visits between infancy and childhood. Ethical approval for this study was given by the London Central NREC (code 06/MRE02/73; 08/H0718/76). Parents/ caregivers of infant participants gave written informed consent before the start of the study. In addition, parental consent, and participant assent (where possible) were obtained at each visit. All methods were performed in accordance with the relevant guidelines and regulations.

In total, 247 participants were included in the cohorts for the study. (Note, an additional 4 infants were recruited (3 females) but excluded, because data on family history of autism for these infants were unavailable^32,33^.) Familial history of autism was assessed at study entry. Infants in the family history (FH) group all had an older sibling with a community clinical diagnosis of autism. Infants without a familial history of autism (no familial history—NFH) group had an older sibling with typical development (also see SI1 2.1). EEG data during social and non-social videos analysed in the current study was recorded at the visit at 14 months of age. We have previously reported on alpha-range connectivity collapsed across conditions in an overlapping sample of 155 infants (in an original cohort ^32^, and a replication cohort^33^).

The EEG assessment was included in the 14-month-old visit when infants were between 13 and 18 months of age. Our final sample of infants with sufficient EEG data for each condition included 26 NFH infants and 75 FH infants. Later outcomes were measured at the visit at 36 months of age. Data for the final sample were missing at the 36-month-old visit for the following measures: age in months (2 NFH and 1 FH infant), MSEL ELC (2 NFH and 2 FH infants), VABS-II (3 NFH and 1 FH infants), and SRS-2 (2 NFH and 6 FH infants).

The included samples were overall representative of cohort as the included and excluded samples showed no difference on the demographics measures (see SI1 2.2.), except that age at the 14-month-old visit for the included NFH sample was higher than for the excluded NFH sample.

### Materials and procedure

Materials and procedures for this study are identical to those previously reported in^32,33^. The following provides a short summary while more details can be found in SI1 2.3.

#### EEG at the 14-month-old visit

Infants watched social videos (two women singing nursery 4 rhymes, 32 s duration), and non-social videos (4 moving toys with or without a hand activating them (41 s and 44 s, resp., see Fig. 1a). One block consisted of these 3 videos presented. This block was then repeated twice (in the same order) with other EEG tasks interspersed, resulting in a total of 9 video presentations. Infants sat on their parents’/caregivers’ lap while the EEG was recorded with a high-density 128 channel EGI electrode system at a sampling rate of 500 Hz, with Netstation EGI software (Electro Geodesics, In., Eugene, USA). EEG recordings were terminated when the infant refused the net or became fussy. Sessions were video recorded for further preprocessing.

#### Behavioural measures

The Mullen Scales of Early Learning (MSEL^80^) were used as a direct assessment of developmental skills at both the 14- and 36-month-old visits. The Early Learning Composite (ELC) score reflects the overall level of cognitive skills. Second, the Vineland Adaptive Behaviour Scale—Second Edition (VABS-II^81,82^) is a parental questionnaire/interview measuring adaptive behaviours. The standard scores (mean 100, and standard deviation 15) were derived for the Communication domain, and the Socialization domain here. Third, the SRS-2^83^ is a parental questionnaire assessing the severity of traits of autism with separate scores available for Social Communication and Interaction (SCI) and Restricted Interests and Repetitive Behaviour (RRB). Lower standardized T-scores reflect fewer autism-related difficulties.

#### EEG pre-processing

Periods of inattention and parental interference during the EEG session were manually coded from the videos using Mangold INTERACT Software (www.mangold-international.com, Mangold, Canada) and excluded from further analyses (SI1 2.3.1). Further pre-processing analyses were performed in MATLAB (MathWorks, Natick, USA) using the FieldTrip toolbox^84^. Continuous EEG data were visually inspected, and artifacts were manually excluded. Electrodes on the outer rim were excluded due to bad quality from muscle artefacts and insufficient contact with the scalp (red diamond in Fig. 1b). Data for 116 clean electrodes were segmented into 1-s epochs with 50% overlap after which another two rounds of data cleaning followed. First, segments were automatically excluded based on thresholds and jumps. Second, we visually inspected that segments to ensure they were artifact-free and excluded any artifacted segments not picked up in previous rounds of artifact rejection. A Fast Fourier Transform (FFT) with a Hanning window was then applied to each epoch using ft_freqanalysis.m (method = ‘mtmfft’, taper = ‘hanning’, output = ‘fourier’, tapsmofrq = 1, keeptrials = ‘yes’). Spectral power was calculated by squaring the absolute FFT values for each epoch and averaging values across epochs before applying the natural log transform to the data (also see SI1 2.3.2. for further details on EEG pre-processing). For spectral power, log transformed channel values were averaged across all electrodes, and left and right frontal, parietal, temporal, and occipital regions as in^34^ (see Fig. 3b, and SI1 2.3.3.). EEG connectivity was measured with the debiased weighted phase lag index (dbWPLI)^78^ for each combination of electrodes (resulting in 116 × 116 connectivity matrix). The dbWPLI reflects the amount of synchronization between different electrode pairs and is calculated from the FFT values and was calculated using the in-house scripts (scripts are available upon reasonable request)^32^. The dbWPLI was chosen here because of its robustness to noise, volume conduction, and variable amounts of artifact-free data (^32,78,85^, SI1 2.3.4.). EEG connectivity measures require a large amount of data which is challenging in infant studies. Previous infant studies used a cut-off of 120 epochs^32,33^, but recent study suggested test–retest reliability for a cut-off of 90 epochs is excellent (ICC = .76)^79^.

We selected 4–5 Hz as our frequency band of interest. This decision was based on (a) the theta band selected by Orekhova et al.^32^ in the previous study on a subsample of the current dataset focusing on oscillations across all videos, (b) a peak around 4 and 5 Hz based on visual inspection of the aggregated frequency spectra (blind to group) for power and connectivity in the current sample, (c) avoiding contamination and overlap with the alpha band examined in the previous studies in the same sample (6–9 Hz^34,39^ and 7–8 Hz^32,33^ (SI1 2.3.5.)). We then averaged spectral power and connectivity across the theta band and across identical numbers of epochs from the social and non-social conditions (SI1 2.3.6.), separately. To keep the sample consistent across the theta power and connectivity analyses, we only included infants with 90 or more clean epochs per condition (social and non-social) in further analyses. This resulted in a sample of 101 infants including 26 NFH infants and 75 FH infants. Figure 1c displays the attrition rates. The numbers of epochs included in the analyses did not differ between groups (M_NFH_ = 130, std = 23, range 91–177, and M_FH_ = 134, std = 29, range 90–233, *t*(99) = − .69, *p* = .493).

### Statistical analyses

For theta spectral power, we used a 2 × 4 × 2 × 2 mixed ANOVA with Condition (Social, Non-Social), Region (Frontal, Temporal, Parietal, Occipital), and Hemisphere (Left, Right) as within-subject factors, and Group (NFH, FH) as between-subject factor in SPSS. We subsequently examined whether other variables may have influenced the results, such as the number of epochs^78,85^, overlap across epochs, and age and cognitive levels^86,87^ (included as covariates in separate GLMs), and sex, as emerging autism traits may differ between sexes^88^ (included as additional factor in a separate GLM).

For theta connectivity, we examined condition modulations at a global level and an individual channel pair level. First, we tested for differences in global connectivity applying a 2 × 2 mixed ANOVA to the global connectivity values (averaged dbWPLI across all channel pairs) with Condition (Social, Non-Social) as within-subject factor, and Group (NFH, FH) as between-subject factor in SPSS. The global connectivity values did not show Gaussian distributions and were therefore first log transformed (after adding .004 as log10 can only be calculated for positive values).

Second, we applied Network Based Statistics (NBS^89^) to the connectivity matrices to test the effect of condition at each individual channel pair. NBS is a nonparametric statistical method that uses permutation testing to correct for multiple comparisons (comparing connectivity between each electrode pair, for more details on the NBS statistical method see SI1 2.4). If a cluster of edges shows a consistent effect, the NBS graphical user interface outputs a network of electrode pairs, or ‘mask’, while otherwise, the NBS output is empty. Hypotheses are specified using GLM. We took a stepped approach and first tested whether connectivity differed between conditions within the whole sample. Next, we repeated this analysis within the separate family history groups to examine connectivity modulations related to family history of autism. The GLM was specified with contrast in both positive and negative directions of the condition effect (t-test, 5000 permutations, significance level 0.05, with Extent, threshold at 3.5, and using exchange blocks for repeated measures in the NBS GUI). Thus, the output provides masks (pairs of channels) that significantly vary in connectivity by condition (either Social > Non-Social, or the reverse). To further explore the direction of the NBS results, we averaged connectivity values across the electrode pairs identified by the NBS mask. We then applied a 2 × 2 mixed ANOVA analyses in SPSS to the masked connectivity values with Condition (Social, Non-Social) as within-subject factor, and Group (NFH, FH) as between-subject factor. As with the global connectivity, masked connectivity values were transformed prior to the ANOVA analyses (adding .0063 and log 10 transformation).

We were furthermore interested whether any condition effects varied with family history group on an individual channel pair level. We used NBS to test for an interaction effect between Condition (Social, Non-Social) and Group (NFH, FH). The same parameters were used for these analyses as in the NBS analyses above (t-test, 5000 permutations, significance level 0.05, with Extent, threshold at 3.5, and using exchange blocks for repeated measures in the NBS GUI).

Third, we tested whether stronger *modulation* by social context of the masked connectivity and overall power (averaged across the 8 regions of interest) was related to fewer subsequent social and communication difficulties at 36 months of age. We ran separate Spearman’s correlations with masked connectivity and overall power differences between conditions (social–non-social) for different measures of social and communication difficulties: VABS-II standard scores for the Socialisation domain and the Communication domain, and SRS-2 T-scores from the SCI domain. Finally, we tested the specificity to social behaviour by testing for correlations between the neural measures and overall developmental skills measured by the MSEL ELC, and restricted and repetitive behaviours measured by the SRS-2 T-scores for the RRB domain. All correlations were tested in SPSS.

As the current analyses were secondary, we performed a post hoc statistical power analyses using G*Power^90^ to confirm we had sufficient power to detect a medium-sized interaction effect between group and condition. With a total sample size of 101 infants and a statistical design with 2 groups and 2 repeated measurements (condition), our likelihood of detecting a medium-sized interaction effect (*f* = .25) was 99%. (Note, other input values were kept on default: alpha = 0.05, correlation among repeated measures = .5, and no correction for non-sphericity).

## Supplementary Information

Supplementary Information.

## Data Availability

The datasets generated and analysed during the current study are not publicly available due to ethical regulations for the nature of these data that contain linked and personally identifiable information. Ethical regulations require we share data with appropriate security arrangements. Data can be requested via formal inquired via the BASIS website (http://www.basisnetwork.org/collaboration-and-project-affiliation/).
