# Supplementary material for: Modulation of EEG theta by naturalistic social content is not altered in infants with family history of autism
Source: Sci Rep. 2022 Dec 1;12:20758. doi: 10.1038/s41598-022-24870-7 (PMC9715667; doi:10.1038/s41598-022-24870-7)
Supplement: Supplementary file 1 — Supplementary Information. [file 41598_2022_24870_MOESM1_ESM.pdf]

Supplementary Information 1: Comparisons between family history groups

Table of Contents

1. Supplementary results ***2***

1.1. Detailed description of spectral power findings **2**

1.2. Control analyses for spectral power findings **3**

1.3. Control analyses for connectivity findings **5**

1.4. Theta power modulations and language abilities **7**

2. Supplementary methods ***8***

2.1. Characterisation of the family history of autism **8**

2.2. Representativeness of the included sample **9**

2.3. EEG preprocessing **10**

2.3.1. Behavioural coding during EEG recording 10

2.3.2. EEG preprocessing 11

2.3.3. Spectral power 12

2.3.4. EEG connectivity 12

2.3.5. Selection of theta band 15

2.3.6. Increasing numbers of epochs for the social and non-social conditions 16

2.4. Network Based Statistics (NBS) **17**

References ***18***

1. Supplementary results

1.1. Detailed description of spectral power findings

In the main manuscript we report on the main findings from our spectral power analyses. Here, we provide a more detailed description of the findings of the significant 3-way interactions with condition: Condition, Region, and Hemisphere (p = .037, η_p^2 = .030); and Region, Hemisphere and Group (p = .026, η_p^2 = .031). We further examined the 3-way interaction between Condition, Region, and Hemisphere by performing 2x2 repeated measures GLMs in the separate regions as in[1]. The occipital region showed higher power during social than non-social videos (p < .0001, η_p^2 = .346), and this difference varied by hemisphere (p < .0001, η_p^2 = .176) with stronger condition differences in the left (p < .0001) than the right occipital region (p < .0001). In the frontal regions, power was higher during social compared to non-social videos (p < .0001, η_p^2 = .275) and higher on the left than the right (p = .002, η_p^2 = .096). In the parietal region, power varied by condition (social > non-social, p < .0001, η_p^2 = .119). The interaction reached a trend (p = .054, η_p^2 = .037), with a stronger condition effect in the left (p < .0001) than in the right parietal area (p = .009). The temporal region only displayed trends: the effect for hemisphere (left > right, p = .069, η_p^2 = .033), and the interaction between condition and hemisphere (p = .071, η_p^2 = .032). Follow-up paired samples t-tests however revealed no differences between condition in either hemisphere (ps ≥ .164).

To follow up on our Region, Hemisphere and Group interaction, we split up the family history groups to examine how the patterns of overall power differed between the groups. In the NFH group, overall power varied across regions (p < .0001, η_p^2 = .664), where power was highest in occipital areas, then frontal and temporal regions which did not differ from each other, and lowest in parietal regions. The interaction between region and hemisphere reached a trend (p = .089, η_p^2 = .083): power was higher in the left than right temporal region (p = .034) but did not differ with laterality in the other regions (ps ≥ .445). There was no difference between hemispheres overall in this group (p = .320, η_p^2 = .040). In the FH group, power differed between all regions (p < .0001, η_p^2 = .707), with highest power in occipital regions, then temporal, then frontal, and then parietal regions. Power in the FH group was overall higher in left than right regions (p = .003, η_p^2 = .112), and this pattern did not vary by region (p = .271, η_p^2 = .017).

1.2. Control analyses for spectral power findings

It is possible that the main findings of theta power modulations have been influenced by confounding factors such as age or numbers of epochs. We repeated the ANOVA analyses for power while including other variables to examine whether significance levels and effect sizes for any of the observed effects changed with these potentially confounding factors. Separate ANOVAs were performed including the number of epochs, difference in percentage of overlap among epochs between conditions, percentage of hand epochs (SI1 2.3.6), age at EEG assessment (in days), and cognitive abilities (Early Learning Composite score of the MSEL) as covariates. We first centred each of the covariates around their mean by subtracting the mean across the whole sample (101 infants) from each infant's individual value[2]. In the last ANOVA, we included biological sex (male, female) as between-subject factor into the model.

Table SI1.1 displays an overview of the results of the control analyses for spectral theta power, reporting on the p-values and effect sizes for the effect described in the main manuscript. The overall pattern of effects and interactions reached significance did not change when including the number of epochs, difference in percentage of overlap among epochs between conditions, percentage of hand epochs, age at EEG assessment, or cognitive abilities. When including sex in the analyses, the pattern of findings changed where the main effect for family history group reached significance (p = .049, η_p^2 = .039), and the 3-way interactions with group and condition no longer reached significance. We repeated the analyses in the separate groups of males and females to further examine these results. In the group of males, overall theta power tended to be increased in NFH males compared to FH males (p = .054, η_p^2 = .078; $_{NFH\text{-}Male}$ = 2.80, se = 0.16; $M_{FH\text{-}Male}$ = 2.46, se = 0.08). The interaction between Condition and Hemisphere reached a trend in the males, but the main effect of Hemisphere (p = .875, η_p^2 = .001) and the 3-way interactions with Hemisphere (p's ≥ .403, η_p^2's = .021) did no longer reach significance. In the group of females, the pattern of effects was similar to the main findings with the exception of the interaction between Condition, Region, and Hemisphere which now reached a trend instead of significance (p = .058, η_p^2 = .048). Possibly, the hemispheric effects are driven by the females in the sample whereas the family history group are driven by the males.

Table SI1.1. Control analyses for theta spectral power

Confounding variable	*Main effects*			
	Condition	*Group*	*Region*	*Hemisphere*
None	*p* < .0001, **η_p^2 = .195**	*p* = .133, η_p^2 = .023	*p* < .0001, **η_p^2 = .646**	*p* = .014, **η_p^2 = .059**
Number of epochs	*p* < .0001, **η_p^2 = .195**	*p* = .165, η_p^2 = .020	*p* < .0001, **η_p^2 = .647**	*p* = .015, **η_p^2 = .059**
Difference in overlap	*p* < .0001, **η_p^2 = .196**	*p* = .134, η_p^2 = .023	*p* < .0001, **η_p^2 = .647**	*p* = .015, **η_p^2 = .059**
Percentage hand epochs	*p* < .0001, **η_p^2 = .190**	*p* = .165, η_p^2 = .020	*p* < .0001, **η_p^2 = .646**	*p* = .013, **η_p^2 = .062**
Age at EEG assessment	*p* < .0001, **η_p^2 = .194**	*p* = .116, η_p^2 = .025	*p* < .0001, **η_p^2 = .646**	*p* = .016, **η_p^2 = .058**
MSEL ELC	*p* < .0001, **η_p^2 = .196**	*p* = .133, η_p^2 = .023	*p* < .0001, **η_p^2 = .646**	*p* = .020, **η_p^2 = .054**
Sex*	*p* < .0001, **η_p^2 = .167**	*p* = .049, **η_p^2 = .039**	*p* < .0001, **η_p^2 = .624**	*p* = .043, **η_p^2 = .042**
In males	*p* = .002, **η_p^2 = .198**	*p* = .054, η_p^2 = .078	*p* < .0001, **η_p^2 = .619**	*p* = .875, η_p^2 = .001
In females	*p* = .002, **η_p^2 = .181**	*p* = .440, η_p^2 = .012	*p* < .0001, **η_p^2 = .655**	*p* = .007, **η_p^2 = .113**

Table SI1.1. (continued) Control analyses for theta spectral power

Confounding variable	*2 and 3-way interaction effects*			
	Con Reg*	*Con*Hemi*	*Con*Reg*Hemi*	*Reg*Hemi*Grp*
None	*p* < .0001, **η_p^2 = .288**	*p* = .002, **η_p^2 = .091**	*p* = .037, **η_p^2 = .030**	*p* = .026, **η_p^2 = .031**
Number of epochs	*p* < .0001, **η_p^2 = .288**	*p* = .002, **η_p^2 = .091**	*p* = .032, **η_p^2 = .029**	*p* = .026, **η_p^2 = .031**
Difference in overlap	*p* < .0001, **η_p^2 = .292**	*p* = .002, **η_p^2 = .091**	*p* = .030, **η_p^2 = .030**	*p* = .027, **η_p^2 = .031**
Percentage hand epochs	*p* < .0001, **η_p^2 = .288**	*p* = .003, **η_p^2 = .089**	*p* = .023, **η_p^2 = .032**	*p* = .026, **η_p^2 = .031**
Age at EEG assessment	*p* < .0001, **η_p^2 = .290**	*p* = .002, **η_p^2 = .093**	*p* = .040, **η_p^2 = .028**	*p* = .027, **η_p^2 = .031**
MSEL ELC	*p* < .0001, **η_p^2 = .292**	*p* = .002, **η_p^2 = .092**	*p* = .037, **η_p^2 = .029**	*p* = .014, **η_p^2 = .035**
Sex*	*p* < .0001, **η_p^2 = .272**	*p* = .004, **η_p^2 = .084**	*p* = .117, η_p^2 = .062	*p* = .062, η_p^2 = .025
In males	*p* < .0001, **η_p^2 = .267**	*p* = .068, η_p^2 = .071	*p* = .403, η_p^2 = .021	*p* = .407, η_p^2 = .021

In females	$p < .0001$, **η_p^2 = .292**	$p = .018$, **η_p^2 = .104**	$p = .058$, $\eta_p^2 = .048$	$p = .047$, **η_p^2 = .050**

* The main effect of sex reached significance: $F(1,97) = 4.83$, $p = .030$, $\eta_p^2 = .047$, where spectral power tended to be stronger for males than females ($M_{Male} = 2.63$, se = 0.09; $M_{Female} = 2.38$, se = 0.07) which is consistent with findings in[1].

Overall patterns of the results remained the same when including the other variables. This suggests that theta power modulations by condition are minimally influenced by technical factors such as numbers of epochs, overlap between epochs, or composition of the non-social condition (percentage epochs from the hand condition), or by demographic factors such as age of assessment and developmental levels. Biological sex appears relate to the pattern of spectral power modulations in different groups, but current sample sizes and small changes in significance values and effect sizes do not allow for strong conclusions (i.e., in the NFH group, $N_{males} = 9$ vs. $N_{females} = 17$, and in the FH group Ns are 39 and 36, resp.).

1.3. Control analyses for connectivity findings

The connectivity findings may have been influenced by confounding factors as well. As with the theta power analyses, we therefore performed control analyses including the other variables: number of epochs, difference in overlapping epochs between conditions, percentage of hand epochs, age in days at the EEG assessment, MSEL ELC at the EEG assessment, and biological sex. We took the same approach as the control analyses for the spectral power findings, using whole-head theta connectivity and social network connectivity as dependent variables instead of theta power (also see section 2.1 of this document).

The results of the control analyses for whole head theta connectivity are presented in Table SI1.2. None of the confounding variables changed the overall pattern of findings: the main effect for Condition remained significant, whereas the main effect for Group and the interaction between Condition and Group remained not significant. The results for the control analyses for social network connectivity are displayed in Table SI1.3. The overall pattern of findings with a significant main effect for Condition only was still present when correcting for the confounding factors.

Table SI1.2. Control analyses for whole-head theta connectivity

Confounding variable	*Effects*		
	Condition	*Group*	*Con*Grp*
None	p < .0001, **η_p^2 = .126**	p = .380, η_p^2 = .008	p = .216, η_p^2 = .015
Number of epochs	p < .0001, **η_p^2 = .126**	p = .380, η_p^2 = .008	p = .216, η_p^2 = .015
Difference in overlap	p < .0001, **η_p^2 = .128**	p = .381, η_p^2 = .008	p = .212, η_p^2 = .016
Percentage hand epochs	p < .0001, **η_p^2 = .128**	p = .394, η_p^2 = .007	p = .200, η_p^2 = .017
Age at EEG assessment	p < .0001, **η_p^2 = .131**	p = .326, η_p^2 = .010	p = .186, η_p^2 = .018
MSEL ELC	p < .0001, **η_p^2 = .126**	p = .438, η_p^2 = .006	p = .216, η_p^2 = .016
Sex*	p = .002, **η_p^2 = .098**	p = .144, η_p^2 = .022	p = .413, η_p^2 = .007

* There was a significant interaction between family history group and sex: $F(1,97)$ = 5.00, p = .028, η_p^2 = .049, with males in the NFH group showing increased whole-head theta connectivity compared to males in the FH group ($F(1,46)$ = 9.40, p = .004, η_p^2 = .170; M_{NFH} = -1.47, se = 0.09; M_{FH} = -1.78, se = 0.04), whereas there are no differences between groups in the females ($F(1,51)$ = 0.27, p = .603, η_p^2 = .005; M_{NFH} = -1.81, se = 0.10; M_{FH} = -1.74, se = 0.07). The main effect of Sex reached a trend ($F(1,97)$ = 3.21, p = .076, η_p^2 = .032), where global connectivity tended to be elevated for males (M = -1.63, se = .07) than for females (M = -1.78, se = 0.05).

Table SI1.3. Control analyses for theta connectivity within the social network

Confounding variable	*Effects*		
	Condition	*Group*	*Con*Grp*
None	p < .0001, **η_p^2 = .273**	p = .681, η_p^2 = .002	p = .863, η_p^2 = 0
Number of epochs	p < .0001, **η_p^2 = .273**	p = .696, η_p^2 = .002	p = .865, η_p^2 = 0
Difference in overlap	p < .0001, **η_p^2 = .278**	p = .679, η_p^2 = .002	p = .873, η_p^2 = 0
Percentage hand epochs	p < .0001, **η_p^2 = .269**	p = .742, η_p^2 = .001	p = .779, η_p^2 = .001
Age at EEG assessment	p < .0001, **η_p^2 = .278**	p = .615, η_p^2 = .003	p = .922, η_p^2 = 0
MSEL ELC	p < .0001, **η_p^2 = .270**	p = .564, η_p^2 = .003	p = .861, η_p^2 = 0
Sex*	p < .0001, **η_p^2 = .252**	p = .766, η_p^2 = .001	p = .258, η_p^2 = .013

* The main effect of Sex was significant: ($F(1,97)$ = 5.35, p = .023, η_p^2 = .052), where social network connectivity tended to be elevated for males (M = -1.50, se = .07) than

for females (M = -1.71, se = 0.06). There was a significant interaction between family history group and sex: $F(1,97) = 6.01$, $p = .016$, $\eta_p^2 = .058$, with males in the NFH group showing increased social network connectivity compared to males in the FH group ($F(1,46) = 7.38$, $p = .009$, $\eta_p^2 = .138$; $M_{NFH} = -1.34$, se = 0.11; $M_{FH} = -1.66$, se = 0.05), whereas there are no differences between groups in the females ($F(1,51) = 0.89$, $p = .351$, $\eta_p^2 = .017$; $M_{NFH} = -1.77$, se = 0.10; $M_{FH} = -1.65$, se = 0.07).

Together, these findings suggest that the theta connectivity modulations across the whole head and within the social network are not related to technical factors such as numbers of epochs, overlap between epochs, or composition of the non-social condition (percentage epochs from the hand condition), or by demographic factors such as age of assessment, developmental levels, and biological sex.

1.4. Theta power modulations and language abilities

One line of research suggests theta oscillations may relate to neural tracking of the speech envelope in the nursery rhymes. It has been suggested that neural tracking during infancy may relate to language acquisition. We therefore tested whether the observed differences in theta power between the social and non-social videos was related to concurrent or later language skills measures by the MSEL. We focused on the Receptive and Expressive language scores assessed at the 14- and 36-month-old visit.

The concurrent language scores were not associated with the theta power difference at 14 months: for MSEL Receptive Language T-scores: $r = -0.06$, $p = .541$, and for MSEL Expressive Language T-scores: $r = 0.02$, $p = .862$. Similarly, later language scores were not related to theta power differences at infancy: MSEL Receptive Language T-scores, $r = 0.03$, $p = .760$, or MSEL Expressive Language T-scores, $r = 0.10$, p = .352.

This suggests that our observed condition differences in theta power at 14 months of age are not related to concurrent or later language skills measured at 3 years of age.

2. Supplementary methods

2.1. Characterisation of the family history of autism

In total, 247 infant participants were entered into the BASIS study (www.basisnetwork.org). Data from 2 previous cohorts were collapsed to increase statistical power. The current study reports on characteristics for the combined dataset. Characteristics for the separate datasets can be found in[3,4].

Familial history of autism was assessed at the time of study entry when the infant participants were younger than 5 months of age. Each of the 170 FH (familial history) infants had a sibling (proband) with a clinical diagnosis of autism and who was older than 3 years of age at the time of study entry. Parents of these siblings completed the Development and Wellbeing Assessment (DAWBA[5]), and Social Communication Questionnaire (SCQ[6]) for the proband. Experienced researchers (TC, GP) subsequently reviewed results of these questionnaires along with medical history of the family (significant medical conditions in the proband or their extended family members) in order to confirm the diagnosis in the proband.

Out of the 170 probands for the FH infants, 135 scored above the cut-off on both the DAWBA and SCQ (≥ 15). 11 scored above cut-off on the DAWBA but not on the SCQ, whereas 17 scored above cut-off on the SCQ but not the DAWBA. One of the questionnaires was missing for 3 probands (missing DAWBA for 2 probands, and SCQ for 1 proband), although scores were above cut-offs for each of the available questionnaires. Finally, for 5 probands none of the questionnaires were available, and assessment of familial history was based on family medical history and information available from the community diagnosis. None of the FH infant participants were excluded from the BASIS study after the familial history assessment.

For the 77 NFH (no family history) infants, familial history of autism was assessed using the SCQ only. None of the probands of these infants scored above the cut-off (for 1 proband, the questionnaire was missing), and no significant medical conditions in first-degree family members were observed. All NFH infants showed typical development at 5 months of age. Infants were recruited via the volunteer database at the Centre for Brain and

Cognitive Development at Birkbeck, University of London, UK. No NFH infants were excluded from the BASIS study.

2.2. Representativeness of the included sample

To test whether the included sample is representative of the tested cohort we compared the included and excluded sample. Results for these analyses are presented in Table SI1.4. The included NFH group was older than the excluded FH group. None of the other tested variables displayed a difference between the included and excluded samples, such as age and developmental levels at the 14- and 36-month-old visit, and behavioural measures of autistic traits (VABS-II, SRS-2, ADI-R, ADOS-2).

Table SI1.4. Demographics and comparisons between infants included and excluded from main analyses

	Group	*Included*	*Excluded*	*Total*
Number of infants	NFH	26	51	77
	FH	75	91	166
	Total	101	142	243[1]
	Group	*Included*	*Excluded*	*Adj. p-values*
Age at 14-month-old visit (days)	NFH[2]	470 (33), 401 – 544	439 (40), 359 – 540	.026
	FH[3]	457 (53), 397 – 576	452 (49), 363 – 551	.520
MSEL at 14-month-old visit [a]	NFH[2]	104 (15), 85 – 133	105 (16), 73 – 154	.981
	FH[2]	95 (21), 65 – 123	95 (20), 49 – 147	.981
Age at 36-month-old visit (months)	NFH[3]	38 (2), 36 – 50	38 (3), 35 – 51	.981
	FH[3]	38 (2), 32 – 53	38 (4), 34 – 45	.981
MSEL at 36-month-old visit [a]	NFH[3]	122 (11), 91 – 137	117 (25), 69 – 147	.981
	FH[3]	106 (36), 49 – 145	107 (35), 49 – 147	.981
VABS-II Communication [b]	NFH[2]	109 (11), 85 – 127	108 (10), 89 – 139	.981
	FH[3]	100 (19), 52 – 125	100 (21), 49 – 127	.981
VABS-II Socialization [c]	NFH[2]	105 (6), 92 – 116	105 (9), 85 – 124	.981
	FH[3]	97 (18), 61 – 118	97 (19), 53 – 116	.981
SRS-2 SCI [d]	NFH[2]	41 (4), 35 – 49	43 (4), 35 – 55	.865
	FH[3]	46 (11), 36 – 89	46 (15), 35 – 84	.981
SRS-2 RRB [e]	NFH[3]	40 (2), 40 – 58	42 (6), 40 – 60	.338
	FH[3]	44 (9), 40 – 104	44 (12), 40 – 90	.981
ADI-R Social Total [f]	NFH[3]	1 (2), 0 – 3	0 (1), 0 – 6	.952
	FH[3]	2 (4), 0 – 25	2 (5), 0 – 19	.981
ADI-R, Com Total [g]	NFH[3]	0 (0), 0 – 3	0 (1), 0 – 4	.981
	FH[3]	2 (5), 0 – 20	2 (5), 0 – 19	.981
ADI-R	NFH[3]	0 (0), 0 – 1	0 (0), 0 – 1	.981

BRI Total [h]	FH[3]	0 (2), 0 – 10	0 (2), 0 – 8	.981
ADOS-2,	NFH[3]	3 (5), 0 – 12	4 (5), 0 – 18	.981
SA Total [i]	FH[3]	3 (7), 0 – 20	3 (4), 0 – 16	.981
ADOS-2	NFH[3]	1 (2), 0 – 5	1 (2), 0 – 4	.981
RRB Total [j]	FH[3]	1 (2), 0 – 6	1 (2), 0 – 6	.981

Comparisons reaching significance are printed in bold (FDR adjusted *p*-values (across all 26 comparisons)). Abbreviations: *NFH* no familial history of autism; *FH* familial history of autism
[1] Data on familial history of autism and outcome were missing for 4 infants in the sample.
[2] Means and standard deviations in parentheses; minimum and maximum values; and p-values for independent samples t-tests.
[3] Medians and interquartile range in parentheses; minimum and maximum values; and p-values for Mann-Whitney U tests.
[a] Mullen Scale for Early Learning (MSEL): Early Learning Composite Standard Score.
[b] Vineland Adaptive Behaviour Scale, Communication domain standard score.
[c] Vineland Adaptive Behaviour Scale, Socialization domain standard score.
[d] Social Responsiveness Scale-2, T-score for the Social Communication and Interaction domain.
[e] Social Responsiveness Scale-2, T-score for the Restricted and Repetitive Behaviours domain.
[f] Autism Diagnostic Interview – Revised, Social Algorithm Total at 36 months.
[g] Autism Diagnostic Interview – Revised, Communication Algorithm Total at 36 months.
[h] Autism Diagnostic Interview – Revised, Behaviours/ Repetitive Interests Algorithm Total 36 months.
[i] Autism Diagnostic Observation Scale – 2, Social Affect Total 36 months.
[j] Autism Diagnostic Observation Scale – 2, Restricted and Repetitive Behaviours Total 36 months.
Missing data:
Age at 14-month-old visit: 2 NFH_{Excl} , and 3 FH_{Excl} infants.
MSEL at 14-month-old visit: 3 NFH_{Excl}, and 3 FH_{Excl} infants.
Age at 36-month-old visit: 2 NFH_{Incl}, 2 NFH_{Excl}, 1 FH_{Incl}, and 1 FH_{Excl} infant.
MSEL at 36-month-old visit: 2 NFH_{Incl}, 2 NFH_{Excl}, 2 FH_{Incl}, and 1 FH_{Excl} infant.
VABS-II: 3 NFH_{Incl}, 3 NFH_{Excl}, 1 FH_{Incl}, and 4 FH_{Excl} infants.
SRS-2 scales: 2 NFH_{Incl}, 3 NFH_{Excl}, 6 $FH_{Incl,}$ and 8 FH_{Excl} infants.
ADI-R domains: 15 NFH_{Incl}, 37 NFH_{Excl}, 2 FH_{Incl}, and 1 FH_{Excl} infant.
ADOS-2 domains: 2 NFH_{Incl}, 2 NFH_{Excl}, 1 FH_{Incl}, and 1 FH_{Excl} infant.

2.3. EEG preprocessing

2.3.1. Behavioural coding during EEG recording

Preprocessing and coding of the data was done by different researchers: the dataset of the first cohort was coded by a research assistant and preprocessed by Elena Orekhova, whereas the dataset of the second cohort was coded and preprocessed by the first author of this manuscript (RH). While the preprocessing scripts and steps for EEG data analyses were identical between cohorts, the coding schemes for behaviour during the EEG session were different. In the analysis process of both cohorts, familial history and later outcome were unknown to the researcher performing the behavioural coding and preprocessing

steps. Data on familial history and later outcome data were matched with the EEG data for further statistical analyses after data cleaning and preprocessing steps were completed.

The recordings from the EEG session were coded for attention and interference. Attention was coded from the first frame the child was looking at the screen to the last frame the child was looking at the screen. Interference was defined as any behaviour that is distracting the infant from looking at the screen that would not show up in the EEG signal and has a minimal duration of 1 second. Interference was coded from the first to the last frame when the behaviour was present. Examples of interference are the following: a parent or the experimenter talking to the infant, pointing to the screen to redirect the infant's attention, or stroking the infant.

During data analysis in the first cohort, additional categories were coded: 1) gross body, head, and arm movements, 2) crying, and 3) smiling[3]. These categories were dropped during data analysis of the second cohort because gross movements are more easily identifiable during visual inspection of the EEG data, and coding of crying or smiling might bias the sample towards infants in a neutral state. Analyses comparing the amounts of data between cohorts revealed that infants in the first cohort were more attentive and provided more artefact-free epochs than those in the second cohort. There were no differences between cohorts in the percentages of interference[4].

2.3.2. EEG preprocessing

The following steps were identical for data from both cohorts. Segments of this text have been published elsewhere [4], but are repeated here for the reader's convenience.

The continuous EEG data were first filtered for visual inspection with a high-pass 1 Hz filter and a 48-52 Hz band stop filter. The segments where the child was not looking at the screen and those where there was interference present were marked at bad in the continuous EEG data. Data were visually inspected and episodes with artefacts from muscles, blinks, movement, or electrodes losing signal were marked as bad data segments. Furthermore, bad channels on the outer side of the net were discarded (E17, E48, E49, E73, E81, E88, E113, E119, E125, E126, E127, and E128) (see Figure 3 in main manuscript). Channels that were bad for individual participants were also marked. The raw unfiltered data for these marked channels were interpolated before using the average reference. The data were then filtered with a high-pass 1 Hz filter.

Data segments that had not been marked as bad were cut into 1-second epochs with 50% overlap. Another round of automatic data cleaning followed. The EEG signals for the epoch were interpolated if the signal exceeded a threshold of 150 mkV or showed a jump of more than 100 mkV in 4 ms. Epochs were only interpolated when these events occurred in less than 15% of the channels. If interpolation failed because of an insufficient amount of neighbours, epochs were rejected from further analyses. A second round of visual artefact rejection was done to ensure the 1-second epochs contained no bad data. 1-second epochs that still contained bad data were rejected.

Fast Fourier Transform (FFT) with a Hanning window was applied to the clean epochs. The complex Fourier values were obtained for each epoch, for each channel, and for each frequency between 0 and 250 Hz (N epochs x 116 channels x 251 frequencies).

2.3.3. Spectral power

Spectral power was calculated by squaring the absolute values of the FFT values for each epoch before calculating the average over trials. The values were log transformed using the log function in Matlab. Theta band power was calculated for the frequency band of interest: 4-5Hz (also see section 2.3.5 in this document). Theta power was averaged across electrodes to extract 1 value for different regions of interest: E19, E20, E23, E24, E27, and E28 for frontal left; E3, E4, E117, E118, E123, and E124 for frontal right; E30, E36, E37, E41, E42, and E47 for left parietal; E87, E93, E98, E103, E104, and E105 for right parietal; E39, E40, E44, and E45 for left temporal; E108, E109, E114, and E115 for right temporal; E60, E65, E66, E67, and E70 for left occipital; and E77, E83, E84, E85, and E90 for right occipital regions (as in[1] and see Figure 3 in main manuscript).

2.3.4. EEG connectivity

EEG connectivity reported in the current study was measured using the dbWPLI, which is a version of the phase lag index. The phase lag index (PLI) measures consistent, non-zero phase lags by quantifying the asymmetry of the distribution of the phase differences between 2 signals [7].

$$PLI = |E\ \{sgn(\Im\{X\})\}|,$$

where I{X} is the imaginary component of the cross-spectrum, and E{.} is the expected value operator [8]. Asymmetry arises when the likelihood of a phase difference between -π and 0 degrees is different from the likelihood of a phase difference between 0 andπ. The PLI

ranges from 0 to 1. A value of 0 reflects no coupling or coupling with a phase difference of 0 or π and *low connectivity*, whereas a value of 1 reflects perfect coupling with a phase difference that is not 0 or π and *very high connectivity*.

The PLI is less sensitive to volume conduction than coherence. Coherence has often been used in other studies [9,10]. Volume conduction arises from common pick up from one source by multiple electrodes and by the spread of the electrical field across the scalp [11]. Connectivity values for electrodes with short distances will be overestimated as a result of the volume conduction. This makes it difficult to disentangle whether the connectivity arose from true connectivity between sources or from volume conduction artefacts.

The PLI assumes that non-zero phase lags are more likely to reflect true connectivity, as volume conduction effects cannot account for non-zero phase lags from a single source. Results from model simulations show that the PLI is less sensitive to the effects of common sources than phase coherence and the imaginary part of coherence. Furthermore, the PLI is less affected by different montages than the phase coherence (PC) when applied to EEG recordings. Lastly, spatial patterns in previous studies showed high PC values over short distances and almost 0 values over long connections. This difference between long and short connections was almost absent for PLI values. This suggests that the PLI is less sensitive to signal spread than PC. By focusing on non-zero phase lag, the PLI is less influenced by volume conduction effects than phase coherence [7].

The PLI is less sensitive to volume conduction and therefore gives a better indication of true connectivity compared to coherence measures. However, the PLI is sensitive to noise when the phase difference lies around 0 or 180°. A small amount of noise can turn a phase lead into a lag and vice versa. The weighted PLI (WPLI) weights the phase lag index values to account for this, and is thus less sensitive to noise than the PLI. The WPLI weights the $sgn(\Im\{X\})$ by the magnitude of the imaginary component $|\Im\{X\}|$. The WPLI is calculated as the following:

$$WPLI = \frac{|E\{\Im\{X\}\}|}{E\{|\Im\{X\}|\}} = \frac{|E\{|\Im\{X\}|sgn(\Im\{X\})\}|}{E\{|\Im\{X\}|\}},$$

where I{X} is the imaginary component of the cross-spectrum, and E{.} is the expected value operator. Phase lags with differences closer to 0 or π will be assigned a very small weight, whereas differences closer to ½ π or -½ π receive the largest weights. Thus, the WPLI is less

affected by small phase lags or leads close to the real axis that are easily turned into leads or lags compared to the PLI.

Both the PLI and WPLI are sensitive to the amount of epochs over which the values are averaged [8]. PLI and WPLI values tend to be overestimated for a small number of epochs. The debiased WPLI (dbWPLI) is another version of the WPLI that less influenced by this bias to the number of epochs. The debiased WPLI is calculated as follows:

$$dbWPLI = \frac{\sum_{j=1}^{N}\sum_{k\neq j}\Im\{X_j\}\Im\{X_{jk}\}}{\sum_{j=1}^{N}\sum_{k\neq j}|\Im\{X_j\}\Im\{X_{jk}\}|}$$

We chose to use the dbWPLI as a measure for functional EEG connectivity in the current study. First, because this measure has been successfully used in previous studies [3,4]. Second, due to the debiasing and weighting methods the dbWPLI measure is more robust in the context of noisy infant data that are unlikely to include a large number of clean epochs. The dbWPLI values were calculated from the FFT values for each epoch, and then averaged across all epochs per individual. The dbWPLI values are organized in a connectivity matrix where each row represents the dbWPLI values between 1 channel and each of the 115 other channels. The connectivity matrix for the dbWPLI is mirrored around the diagonal as the dbWPLI measures undirected connectivity.

Different metrics can be derived from the connectivity matrices. First, a connectivity matrix for the frequency band of interest is obtained by averaging the connectivity matrices across frequencies. Second, averaging all values under the diagonal then gives a global dbWPLI value. Connectivity values for each channel are calculated by averaging the values across the columns excluding the values on the diagonal, e.g. ($dbWPLI^{Ch1-Ch2}$ + $dbWPLI^{Ch1-Ch3}$ + … + $dbWPLI^{Ch1-Ch116}$)/115. Third, a mask can be applied to the connectivity matrices to calculate the average connectivity across selected connections, for example the connections that showed increased connectivity in the social condition compared to the non-social condition in the NBS analyses. Connections for electrode pairs that were not included in the mask were set to 0, whereas values for connections included in the mask were unaltered. Global connectivity values for connections included in a specific mask were calculated by averaging all non-zero values. The raw connectivity matrices for the theta frequency, global connectivity across all connections, and global connectivity across selected connections from a mask were used for further statistical analyses discussed in the main text.

2.3.5. Selection of theta band

In the current analyses, we focused on the theta band selecting 4-5Hz as our frequency band of interest. We wanted to confirm that the frequency spectra for power and connectivity displayed a peak in this frequency range. We therefore explored the spectra in our initial exploratory analyses. We included all infants that contributed to the previous connectivity studies (N_{Total} = 155)[3,4]. Both global power (averaged across all channels) and connectivity (averaged across all possible channel pairs) were calculated for each infant for each of the three conditions, separately: social, hand, and toy condition. We then visually inspected these plots (Figure SI1.1). Peaks were indeed more apparent in the connectivity than the power spectra. Based on the previous study and while avoiding overlap from the alpha frequency range (6-9Hz[1,12] and 7-8Hz[3,4]), we defined our theta frequency band from 4 to 5 Hz.

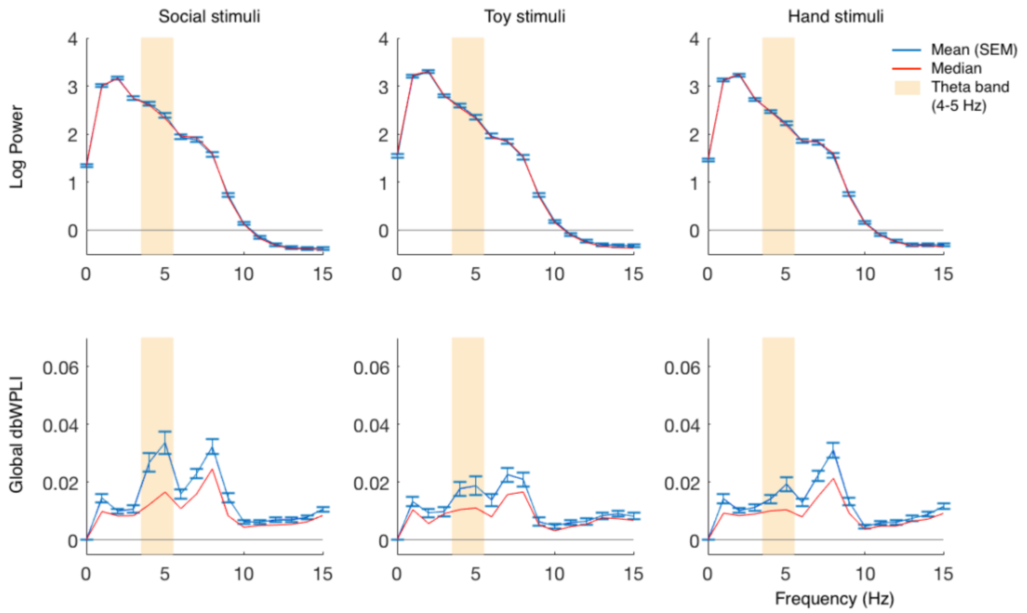

Figure SI1.1. Frequency spectra for power and EEG connectivity
Top row displays log power values, while bottom row displays global dbWPLI values. Different columns represent spectra for the 3 different conditions: social condition (left), toy condition (middle), and hand condition (right). Blue lines represent average values with standard errors of measurements, and red lines median values. The orange rectangle marks the 4 to 5 Hz frequency band. Data are averaged across 155 infants, where data were missing for 1 infant for the toy condition, and for 1 infant for the hand condition.

2.3.6. Increasing numbers of epochs for the social and non-social conditions

Differences in attention to screen and different video lengths for the different conditions resulted in unequal numbers of artefact-free trials for the social (32sec), hand (41sec), and toy (44sec) condition. In addition, a good, reliable estimate of dbWPLI connectivity requires at least 90 epochs per condition[13]. We took several measures in our pre-processing pipeline in order to optimise the data available to us. First, we segmented our continuous data into overlapping epochs with 50% overlap.

Second, we decided to collapse data from the toy and hand conditions into one non-social condition, since this would increase the number of participants included, and thereby increase our statistical power. For each infant, FFT values from different epochs were randomly selected from different conditions while ensuring that a) the amounts of epochs in the social and non-social conditions were matched within each participant, and b) data for the non-social condition included even proportions of epochs from the toy and hand condition where possible (e.g. $N_{Social\ epochs} = ½\ N_{Toy\ epochs} + ½\ N_{Hand\ epochs}$). This method was applied to ensure that epochs were selected from different sections across the EEG session rather than selecting epochs from the beginning of the session only, and in order to rule out any effects of different data amounts between epochs.

These decisions allowed us to include more infants into our sample, which increased our statistical power. However, one might argue that results may relate to these decisions. We therefore calculated some measures that reflect these preprocessing decisions. For the overlap, we calculated the percentage of epochs that overlapped with 1 or 2 other epochs for each condition and the difference in this overlap percentage between conditions. This measure can also be interpreted as a proxy for attention to the videos where higher percentages of overlap reflect data from longer continuous segments and thus higher levels of attention. Next, we calculated the proportion of hand epochs included in the non-social condition investigated in the main text. We calculated the percentage of hand epochs in the non-social condition as follows: $N_{Hand\ epochs}/\ N_{Non\text{-}social\ epochs} * 100$. These measures were used in the ANOVAs for power and connectivity as covariates to examine whether these preprocessing decisions may have influenced our main findings.

2.4. Network Based Statistics (NBS)

In addition to global levels, we examined modulations of social context at the level of individual connections. For these analyses we used the Network Based Statistics program (NBS)[14]. Network Based Statistics (NBS) uses permutation testing to test for differences in networks embedded in connectivity matrices[14]. The advantage of permutation testing is that it avoids the multiple comparisons problem. The multiple comparisons problem arises when values for each connection pair are tested in large networks and the probability of a false positive, or Type I error, increases with the increasing number of tests that are being done. Permutation testing works in 4 steps: 1) the statistical test of the null-hypothesis is tested for every connection in the connectivity matrix A; 2) a chosen test-statistic threshold defines supra-threshold connections; 3) clusters are identified from the supra-threshold connections are a close in the topological space, these clusters are called components; 4) a FWER (family wise error rate)-corrected p-value is calculated for each component with permutation testing.

Permutation testing assumes that if there is no difference between groups or conditions, the data belonging to each group or condition can be randomly assigned to a different group or condition without changing the test-statistic[15]. If there would be a difference between the groups or conditions, the test-statistic would be different when the data are randomly assigned to a different group or condition. With each permutation, the data are randomly assigned to different groups or conditions. Then, steps 1 through 3 from the NBS are repeated and the size of the largest component is saved for each permutation. Repeating this permutation process a thousand times creates a null distribution of sizes for the largest component if the null hypothesis was true.

Finally, the size of the component for the actual data is compared with the null distribution obtained during the permutation testing. The FWER-corrected p-value for the actual data is calculated as the percentage of permutations for which the largest component was the same or greater than the size of the component in the actual data[16].

References

1. Jones, E. J. H., Venema, K., Lowy, R., Earl, R. K. & Webb, S. J. Developmental changes in infant brain activity during naturalistic social experiences. *Dev. Psychobiol.* **57**, 842–853 (2015).
2. Van Breukelen, G. J. P. & Van Dijk, K. R. A. Use of covariates in randomized controlled trials. *J. Int. Neuropsychol. Soc.* **13**, 903–904 (2007).
3. Orekhova, E. V. *et al.* EEG hyper-connectivity in high-risk infants is associated with later autism. *J. Neurodev. Disord.* **6**, 1–11 (2014).
4. Haartsen, R. *et al.* Functional EEG connectivity in infants associates with later restricted and repetitive behaviours in autism; a replication study. *Transl. Psychiatry* **9**, (2019).
5. Goodman, R., Ford, T., Richards, H., Gatward, R. & Meltzer, H. The Development and Well-Being Assessment: Description and Initial Validation of an Integrated Assessment of Child and Adolescent Psychopathology. *J. Child Psychol. Psychiatry* **41**, 645–655 (2000).
6. Rutter, M., Bailey, A. & Lord, C. *The Social Communication Questionnaire*. (Western Psychological Services, 2003).
7. Stam, C. J., Nolte, G. & Daffertshofer, A. Phase lag index: Assessment of functional connectivity from multi channel EEG and MEG with diminished bias from common sources. *Hum. Brain Mapp.* **28**, 1178–1193 (2007).
8. Vinck, M., Oostenveld, R., Van Wingerden, M., Battaglia, F. & Pennartz, C. M. a. An improved index of phase-synchronization for electrophysiological data in the presence of volume-conduction, noise and sample-size bias. *Neuroimage* **55**, 1548–1565 (2011).
9. Righi, G., Tierney, A. L., Tager-Flusberg, H. B. & Nelson, C. A. Functional connectivity in the first year of life in infants at risk for autism spectrum disorder: An EEG study. *PLoS One* **9**, 1–8 (2014).
10. Keehn, B., Vogel-Farley, V., Tager-Flusberg, H. B. & Nelson, C. A. Atypical hemispheric specialization for faces in infants at-risk for Autism Spectrum Disorder. *Autism Res.* **8**, 187–198 (2015).
11. Cohen, M. X. *Analizing Neural Time Series Data: Theory and Practise*. (MIT Press, 2014).
12. van der Velde, B., White, T. & Kemner, C. The emergence of a theta social brain network during infancy. *Neuroimage* **240**, 118298 (2021).
13. Haartsen, R., van der Velde, B., Jones, E. J. H., Johnson, M. H. & Kemner, C. Using multiple short epochs optimises the stability of infant EEG connectivity parameters. *Sci. Rep.* **10**, 12703 (2020).
14. Zalesky, A., Fornito, A. & Bullmore, E. T. Network-based statistic: Identifying differences in brain networks. *Neuroimage* **53**, 1197–1207 (2010).
15. Maris, E. & Oostenveld, R. Nonparametric statistical testing of EEG- and MEG-data. *J. Neurosci. Methods* **164**, 177–190 (2007).
16. Zalesky, A. Reference Manual for NBS Connectome (v1.2). (2012).

Supplementary Information 2: Comparisons between outcomes

Table of Contents

1. Supplementary methods ***2***

1.1. Characterisation of developmental outcome at 36 months of age **2**

1.2. Characterisation of the outcome groups **3**

2. Supplementary results ***8***

2.1. Spectral power modulations by social context **8**

2.1.1. Control analyses for spectral power findings 11

2.2. Connectivity modulations by social context **14**

2.2.1. Control analyses for connectivity findings 15

References ***18***

1. Supplementary methods

1.1. Characterisation of developmental outcome at 36 months of age

Developmental outcome for the participants was assessed at 36 months of age using a range of measures, including parental interviews, behavioural observations, and parental questionnaires. We assessed developmental outcomes at 36 months of age as assessments at this age are more sensitive than at 24 months[1]. Autism symptoms were assessed using the Autism Diagnostic Interview – Revised (ADI-R[2]), and the Autism Diagnostic Observation Schedule – Generic / 2 (ADOS-G / ADOS-2[3,4]). The ADI-R is a semi-structured interview, which measures the severity of autism symptoms in the target individual. In this study, the parental interview was used to measure difficulties with social communication and interactions, and restricted and repetitive behaviours in the young participants. The ADOS-G/2 is an observational assessment providing a direct measure of autism symptoms in the target individual. The ADOS-G was used in the first cohort, while the ADOS-2 was used in the second cohort of the BASIS study. For participants with ADOS-G data, scores were recalculated in accordance with the ADOS-2 guidelines to facilitate comparison between groups. Finally, the SCQ was used to assess the severity of autism symptoms in the toddlers.

A team of experienced researchers led the clinical assessments and decisions in both cohorts (GP, TC). After the 36-month-old visit, these researchers reviewed all information from the ADI-R, ADOS, and SCQ, in addition to the results from the MSEL and VABS-II mentioned in the main text, to determine the developmental outcome of each FH participant. Data for this visit were missing for 4 out of 170 FH infants. 34 FH infants met criteria for autism (FH-autism group), whereas 88 FH infants showed typical development at 36 months of age (FH-TD group). 44 infants did not meet criteria for autism but did display some developmental atypicalities at toddlerhood (FH-Other group): a) scoring above the ADI-R and/or ADOS cut-off for autism (N = 26), b) scoring below 1.5 SD below the population mean on the MSEL Composite scale (< 77.5), Expressive Language subscale, or Receptive Language subscale (< 35) (N = 11), or c) scoring in accordance with both a and b (N = 7). In the current outcome analyses, the FH-autism and FH-Other groups were collapsed into one FH-noTD group to even out group sizes and male : female ratios. Note, no toddlers in the NFH group met criteria for autism, and these were thus analysed as a group.

1.2. Characterisation of the outcome groups

The final sample consisted of 101 infants: 26 NFH infants, 38 FH-TD infants, and 37 FH-noTD infants. The following tables display the attrition rates (Table SI2.1), the demographics (Table SI2.2), and the clinical characterisation (Table SI2.3) for the different groups in the outcome sample.

Table SI2.1. Overview of attrition of EEG data and amount of data included

Attrition rates

	NFH	*FH-TD*	*FH-noTD*	*Total*
Number of participants in cohort (female)	77 (42)	88 (53)	78 (29)	247 (127)[1]
No EEG data available[2]	8 (5)	7 (3)	7 (1)	22 (9)
Insufficient amount of artifact-free EEG epochs[3]	43 (20)	43 (25)	34 (17)	120 (62)
Clean EEG data	26 (17)	38 (25)	37 (11)	101 (53)

Amount of data included in final samples

	NFH	*FH-TD*	*FH-noTD*	*Test statistic*
Number of EEG epochs included per condition (identical for both conditions)	129 (33)[4]<br>91 – 177	138 (40)<br>90 – 233[5]	132 (53)<br>90 – 166	$H(2) = 1.40$,<br>$p = .497$

[1] Outcome data were not available for 4 infants (3 females) which were not further included in the analyses. [2] Due to net refusal, no visit, or equipment failure. [3] At least 90 artifact-free 1-second epochs in both the social and non-social conditions. [4] Medians and interquartile range in parentheses; minimum and maximum values; and results for non-parametric tests for group (NFH, FH-TD, FH-noTD). [5] 2 FH-TD infants viewed the videos more than 3 times resulting in more than the expected 191 epochs.

The outcome groups differed on each of the tested domains for the clinical measures: for the ADI-R Social Total algorithm, the Communication Total algorithm, and the Behaviour/ Repetitive Interests algorithm, and for the ADOS-2 Social Affect algorithm, and the Restricted and Repetitive Behaviour algorithm (*p*'s < .0001). The FH-noTD group exhibited higher scores than the NFH and FH-TD group on all tested subscales of the ADI-R and ADOS subscales. The FH-TD group displayed higher scores compared to the NFH group on the ADI-R Communication Total algorithm, but lower scores than NFH group on the ADOS-2 Social Affect algorithm. The NFH and FH-TD groups displayed similar scores on the other tested scales.

Table SI2.3. Demographics of the sample for comparisons between the social and non-social condition.

	NFH	*FH-TD*	*FH-noTD*	*Test statistic*
Number of participants (female)	26 (17) [1]	38 (25)	37 (11)	**$\chi^2(2) = 12.37$, $p = .002$**
Age at 14-month-old visit (days)	466 (46) [2]<br>401 – 544	458 (52)<br>397 – 547	451 (55)<br>409 – 576	$H(2) = 1.87$, $p = .392$
MSEL ELC [a] at 14-month-old visit	104 (15) [3]<br>85 – 133	99 (13)<br>71 – 121	92 (15)<br>65 – 123	**$F(2,98) = 5.04$, $p = .008$**<br>NFH > FH-noTD
Age at 36-month-old visit (months) [4]	38 (2) [3]<br>36 – 50	39 (3)<br>33 – 53	38 (2)<br>32 – 41	$H(2) = 3.17$, $p = .205$
MSEL ELC [a] at 36-month-old visit [5]	122 (11)[3]<br>91 – 137	119 (24)<br>81 – 142	92 (29)<br>49 – 145	**$H(2) = 32.74$, $p < .0001$**<br>(NFH = FH-TD) > FH-noTD
VABS-II Communication [b, 6] at 36-month-old visit [5]	109 (11) [2]<br>85 – 127	106 (11)<br>85 – 125	91 (14)<br>52 – 125	**$F(2,94) = 20.71$, $p < .0001$**<br>(NFH = FH-TD) > FH-noTD
VABS-II Socialization [c, 6] at 36-month-old visit [5]	105 (6) [2]<br>92 – 116	102 (8)<br>85 – 118	88 (12)<br>61 – 114	**$F(2,94) = 31.25$, $p < .0001$**<br>(NFH = FH-TD) > FH-noTD
SRS-2 SCI [d, 7] at 36-month-old visit [5]	40 (8) [3]<br>35 – 49	44 (6)<br>36 – 80	47 (23)<br>37 – 89	**$H(2) = 16.71$, $p < .0001$**<br>NFH < FH-TD < FH-noTD
SRS-2 RRB [e, 7] at 36-month-old visit [5]	40 (2) [3]<br>40 – 58	44 (6)<br>40 – 66	45 (28)<br>40 – 104	**$H(2) = 18.04$, $p < .0001$**<br>NFH < (FH-TD = FH-noTD)

Significant comparisons (at the α level =.05) are printed in bold.
[1] Pearson Chi-Square with asymptotic significance values (2-sided).
[2] Medians and interquartile range in parentheses; minimum and maximum values; and results for non-parametric tests between groups.
[3] Means and standard deviations in parentheses; minimum and maximum values; and results for parametric tests between groups.
[4] Age at 36-mo-visit was missing for 2 NFH, and 1 FH-TD infant.
[5] Missing data for 2 NFH, 1 FH-TD, and 1 FH-noTD infant.
[6] Missing data for 3 NFH, and 1 FH-TD infant.
[7] Missing data for 2 NFH, 3 FH-TD and 3 FH-noTD infants.
[a] Mullen Scale for Early Learning Early Learning Composite Standard Score.
[b] Vineland Adaptive Behaviour Scale, Communication domain standard score.
[c] Vineland Adaptive Behaviour Scale, Socialization domain standard score.
[d] Social Responsiveness Scale-2, T-score for the Social Communication and Interaction domain.
[e] Social Responsiveness Scale-2, T-score for the Restricted and Repetitive Behaviours domain.

Table SI2.3. Clinical information of the sample for comparisons between the social and non-social condition.

	NFH	*FH-TD*	*FH-noTD*	*Test statistic*
ADI-R Social Total [1, a]	1 (2)<br>0 – 3	1 (2)<br>0 – 11	4 (9)<br>0 – 25	*H(2)* = 27.75,<br>***p* < .0001**<br>FH-noTD > (NFH = FH-TD)
ADI-R, Communication Total [1, b]	0 (0)<br>0 – 3	1 (3)<br>0 – 5	6 (9)<br>0 – 20	*H(2)* = 27.75,<br>***p* < .0001**<br>FH-noTD > FH-TD > NFH
ADI-R BRI Total [1, c]	0 (0)<br>0 – 1	0 (0)<br>0 – 3	2 (5)<br>0 – 10	*H(2)* = 31.69,<br>***p* < .0001**<br>FH-noTD > (NFH = FH-TD)
ADOS-2, SA Total [2, d]	3 (5)<br>0 – 12	1 (1)<br>0 – 5	8 (7)<br>0 – 20	*H*(2) = 45.97,<br>***p* < .0001**<br>FH-noTD > NFH > FH-TD
ADOS-2 RRB Total [2, e]	1 (2)<br>0 – 5	1 (1)<br>0 – 3	2 (3)<br>0 – 6	*H*(2) = 18.88,<br>***p* < .0001**<br>FH-noTD > (NFH = FH-TD)

Comparisons reaching significance are printed in bold ($p < .05$).
Medians and interquartile range in parentheses; minimum and maximum scores; and results for non-parametric tests for Group (NFH, FH-TD, FH-noTD). Abbreviations: *NFH* no familial history of autism; *FH* familial history of autism; *FH-TD* familial history of autism – typical developing outcome; *FH-noTD* familial history of autism – atypical outcome (autism or other).
[1] Missing data for 15 NFH, 1 FH-TD and 1 FH-noTD infant.
[2] Missing data for 2 NFH and 1 FH-TD infant.
[a] Autism Diagnostic Interview – Revised, Social Algorithm Total at 36 months.
[b] Autism Diagnostic Interview – Revised, Communication Algorithm Total at 36 months.
[c] Autism Diagnostic Interview – Revised, Behaviours/ Repetitive Interests Algorithm Total 36 months.
[d] Autism Diagnostic Observation Scale – 2, Social Affect Total 36 months.
[e] Autism Diagnostic Observation Scale – 2, Restricted and Repetitive Behaviours Total 36 months.

In order to examine whether the included sample was representative of the different groups, we compared the included and excluded groups of infants within the NFH, FH-TD, and FH-noTD groups on each of the demographic variables. These are represented in Table SI2.4.

The results of these analyses showed a difference in ages at the 14-month-old visit in the NFH group where included infants were older than excluded infants (p = .039). At the visit at 36 months of age, FH-TD toddlers whose EEG measures at 14 months were included displayed lower scores for the ADOS-2 Social Affect Algorithm Total compared to FH-TD

toddlers who were excluded from the main analyses. No other contrasts reached significance.

These findings suggest that the included sample of infants is largely representative of the complete sample, with the exception of age at the 14-month-old visit in the NFH group and the ADOS-2 Social Affect scores at 36 months in the FH-TD group. Older infants possibly had larger amounts of artefact-free EEG data. For example, because they are less easily distracted and thus more attentive to the videos, they are moving around less, or they are more tolerant of the EEG net than younger infants. As for the differences in included and excluded FH-TD samples for measures at 36 months of age, it is possible that subtle individual differences in the FH-TD group in social skills lead to differences in attention to the dynamic videos or response to attempts from the parent or experimenter to redirect the attention to the screen at the infant EEG assessment.

Table SI2.4. Demographics and comparisons between infants included and excluded from main analyses

	Group	*Included*	*Excluded*	*Total*
Number of infants	NFH	26	51	77
	FH-TD	38	50	88
	FH-noTD	37	41	78
	Total	101	142	243[1]
	Group	*Included*	*Excluded*	*Adjusted p-values*
Age at 14-month-old visit (days)	NFH[2]	470 (33), 401 – 544	439 (40), 359 – 540	**.039**
	FH-TD[2]	467 (36), 397 – 547	449 (43), 363 – 548	.320
	FH-noTD[3]	451 (55), 409 – 576	449 (45), 365 – 551	.854
MSEL at 14-month-old visit [a]	NFH[2]	104 (15), 85 – 133	105 (16), 73 – 154	.936
	FH-TD[2]	99 (13), 71 – 121	100 (16), 63 – 147	.936
	FH-noTD[2]	92 (15), 65 – 123	89 (17), 49 – 121	.854
Age at 36-month-old visit (months)	NFH[3]	38 (2), 36 – 50	38 (3), 35 – 51	.854
	FH-TD[3]	39 (3), 33 – 53	38 (3), 36 – 45	.936
	FH-noTD[3]	38 (2), 32 – 41	38 (4), 34 – 43	.936
MSEL at 36-month-old visit [a]	NFH[2]	120 (11), 91 – 137	115 (18), 69 – 147	.755
	FH-TD[3]	119 (24), 81 – 142	112 (23), 79 – 140	.302
	FH-noTD[2]	91 (24), 49 – 145	91 (28), 49 – 147	.936
VABS-II Communication [b]	NFH[2]	109 (12), 85 – 127	108 (10), 89 – 139	.936
	FH-TD[2]	106 (11), 85 – 125	101 (10), 67 – 127	.299
	FH-noTD[2]	91 (14), 52 – 125	92 (18), 49 – 123	.936

VABS-II Socialization [c]	NFH[2]	105 (6), 92 – 116	105 (9), 85 – 124	.936
	FH-TD[2]	102 (8), 85 – 118	101 (9), 79 – 116	.936
	FH-noTD[2]	88 (12), 61 – 114	87 (15), 53 – 110	.936
SRS-2 SCI [d]	NFH[2]	41 (4), 35 – 49	43 (4), 35 – 55	.711
	FH-TD[3]	44 (6), 36 – 80	43 (8), 35 – 81	.854
	FH-noTD[3]	47 (23), 37 – 89	52 (26), 36 – 84	.854
SRS-2 RRB [e]	NFH[3]	40 (2), 40 – 58	42 (6), 40 – 60	.711
	FH-TD[3]	44 (6), 40 – 66	44 (6), 40 – 82	.936
	FH-noTD[3]	45 (28), 40 – 104	50 (33), 40 – 90	.936
ADI-R Social Total [f]	NFH[3]	1 (2), 0 – 3	0 (1), 0 – 6	.714
	FH-TD[3]	1 (2), 0 – 11	1 (3), 0 – 7	.854
	FH-noTD[3]	4 (9), 0 – 25	5 (11), 0 – 19	.936
ADI-R, Com Total [g]	NFH[3]	0 (0), 0 – 3	0 (1), 0 – 4	.936
	FH-TD[3]	1 (3), 0 – 5	1 (3), 0 – 11	.854
	FH-noTD[3]	6 (9), 0 – 20	5 (11), 0 – 19	.936
ADI-R BRI Total [h]	NFH[3]	0 (0), 0 – 1	0 (0), 0 – 1	.936
	FH-TD[3]	0 (0), 0 – 3	0 (1), 0 – 3	.711
	FH-noTD[3]	2 (5), 0 – 10	2 (6), 0 – 8	.936
ADOS-2, SA Total [i]	NFH[3]	3 (5), 0 – 12	4 (5), 0 – 18	.954
	FH-TD[3]	1 (1), 0 – 5	2 (3), 0 – 7	**.039**
	FH-noTD[3]	8 (7), 0 – 20	6 (8), 0 – 16	.711
ADOS-2 RRB Total [j]	NFH[3]	1 (2), 0 – 5	1 (2), 0 – 4	.854
	FH-TD[3]	1 (1), 0 – 3	1 (2), 0 – 5	.854
	FH-noTD[3]	2 (3), 0 – 6	2 (3), 0 – 6	.936

Comparisons reaching significance are printed in bold (FDR adjusted *p*-values (across all 39 comparisons)). Abbreviations: *NFH* no familial history of autism; *FH* familial history of autism; *FH-TD* familial history of autism – typical developing outcome; *FH-noTD* familial history of autism – atypical outcome (autism or other).

[1] Data on familial history of autism and outcome were missing for 4 infants in the sample.

[2] Means and standard deviations in parentheses; minimum and maximum values; and p-values for independent samples t-tests.

[3] Medians and interquartile range in parentheses; minimum and maximum values; and p-values for Mann-Whitney U tests.

[a] Mullen Scale for Early Learning (MSEL): Early Learning Composite Standard Score.

[b] Vineland Adaptive Behaviour Scale, Communication domain standard score.

[c] Vineland Adaptive Behaviour Scale, Socialization domain standard score.

[d] Social Responsiveness Scale-2, T-score for the Social Communication and Interaction domain.

[e] Social Responsiveness Scale-2, T-score for the Restricted and Repetitive Behaviours domain.

[f] Autism Diagnostic Interview – Revised, Social Algorithm Total at 36 months.

[g] Autism Diagnostic Interview – Revised, Communication Algorithm Total at 36 months.

[h] Autism Diagnostic Interview – Revised, Behaviours/ Repetitive Interests Algorithm Total 36 months.

[i] Autism Diagnostic Observation Scale – 2, Social Affect Total 36 months.

[j] Autism Diagnostic Observation Scale – 2, Restricted and Repetitive Behaviours Total 36 months.

Missing data:
Age at 14-month-old visit: 2 NFH_{Excl} , 2 FH-TD_{Excl} , and 1 FH-$noTD_{Excl}$ infant.
MSEL at 14-month-old visit: 3 NFH_{Excl}, 2 FH-TD_{Excl} and 1 FH-$noTD_{Excl}$ infant.
Age at 36-month-old visit: 2 NFH_{Incl}, 2 NFH_{Excl}, 1 FH-TD_{Incl}, and 1 FH-$noTD_{Excl}$ infant.
MSEL at 36-month-old visit: 2 NFH_{Incl}, 2 NFH_{Excl}, 1 FH-TD_{Incl}, 1 FH-$noTD_{Incl}$ and 1 FH-$noTD_{Excl}$ infant.
VABS-II: 3 NFH_{Incl}, 3 NFH_{Excl}, 1 FH-TD_{Incl}, 1 FH-TD_{Excl}, and 3 FH-$noTD_{Excl}$ infants.
SRS-2 scales: 2 NFH_{Incl}, 3 NFH_{Excl}, 3 FH-$TD_{Incl,}$ 1 FH-TD_{Excl}, 3 FH-$noTD_{Incl,}$ 7 FH-$noTD_{Excl}$ infants.
ADI-R domains: 15 NFH_{Incl}, 37 NFH_{Excl}, 1 FH-TD_{Incl}, 1 FH-$noTD_{Incl}$, and 1 FH-$noTD_{Excl}$ infant.
ADOS-2 domains: 2 NFH_{Incl}, 2 NFH_{Excl}, 1 FH-TD_{Incl}, and 1 FH-$noTD_{Excl}$ infant.

2. Supplementary results

2.1. Spectral power modulations by social context

Topoplots for the different conditions and condition differences for each outcome group are displayed in Figure SI2.1. Theta power was higher for the social than the non-social condition ($p < .0001$, $\eta_p^2 = .209$). Theta power varied with region ($p < .0001$, $\eta_p^2 = .691$), showing highest values for occipital, then temporal and frontal regions, and lowest values for parietal regions. Theta power was higher in the left than right hemisphere ($p < .0001$, $\eta_p^2 = .079$).

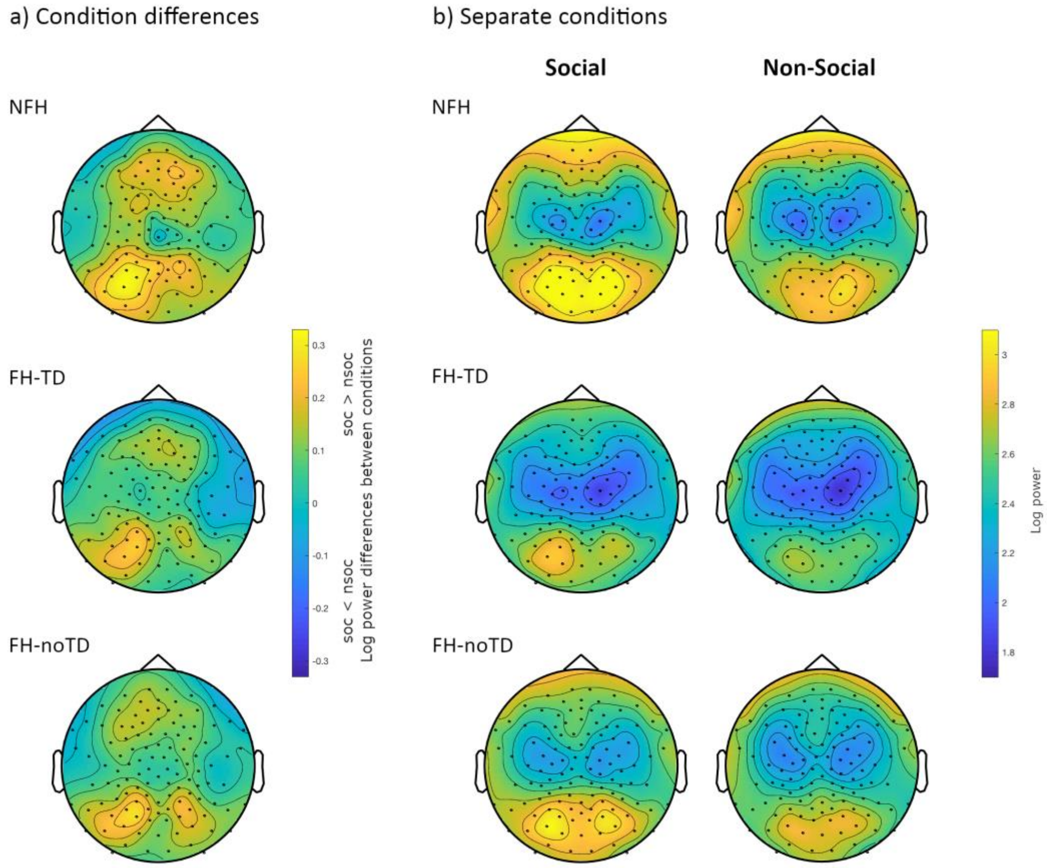

Figure SI2.1. Theta topoplots
a) Topoplots displaying the theta power differences between the social and non-social conditions within the NFH (top row), NH-TD (middle row), and FH-noTD (bottom row) group. b) Topoplots displaying the theta power during the social (left column) and non-social (right column) condition within the NFH (top row), NH-TD (middle row), and FH-noTD (bottom row) group.

There were significant interactions between Condition and Region ($p < .0001$, η_p^2 = .334); Condition and Hemisphere ($p < .0001$, η_p^2 = .125); Condition, Region and Hemisphere ($p = .029$, η_p^2 = .032); and Condition, Region, Hemisphere and Group ($p = .029$, η_p^2 = .046). Because our interest was in condition effects, we calculated the difference in power between conditions (Social – Non-Social). We then examined the pattern of condition effects using 3 separate 4x2 ANOVAs with Region (Frontal, Temporal, Parietal, Occipital) and Hemisphere (Left, right) as within-subject factors in each of the 3 group (Figure SI2.2). In the NFH group, there was an effect of Region ($p < .0001$, η_p^2 = .320) where increases in power during the social versus non-social condition were equally strong in the frontal and occipital regions. These condition differences were stronger than in the temporal and parietal regions, where condition differences did not differ from each other.

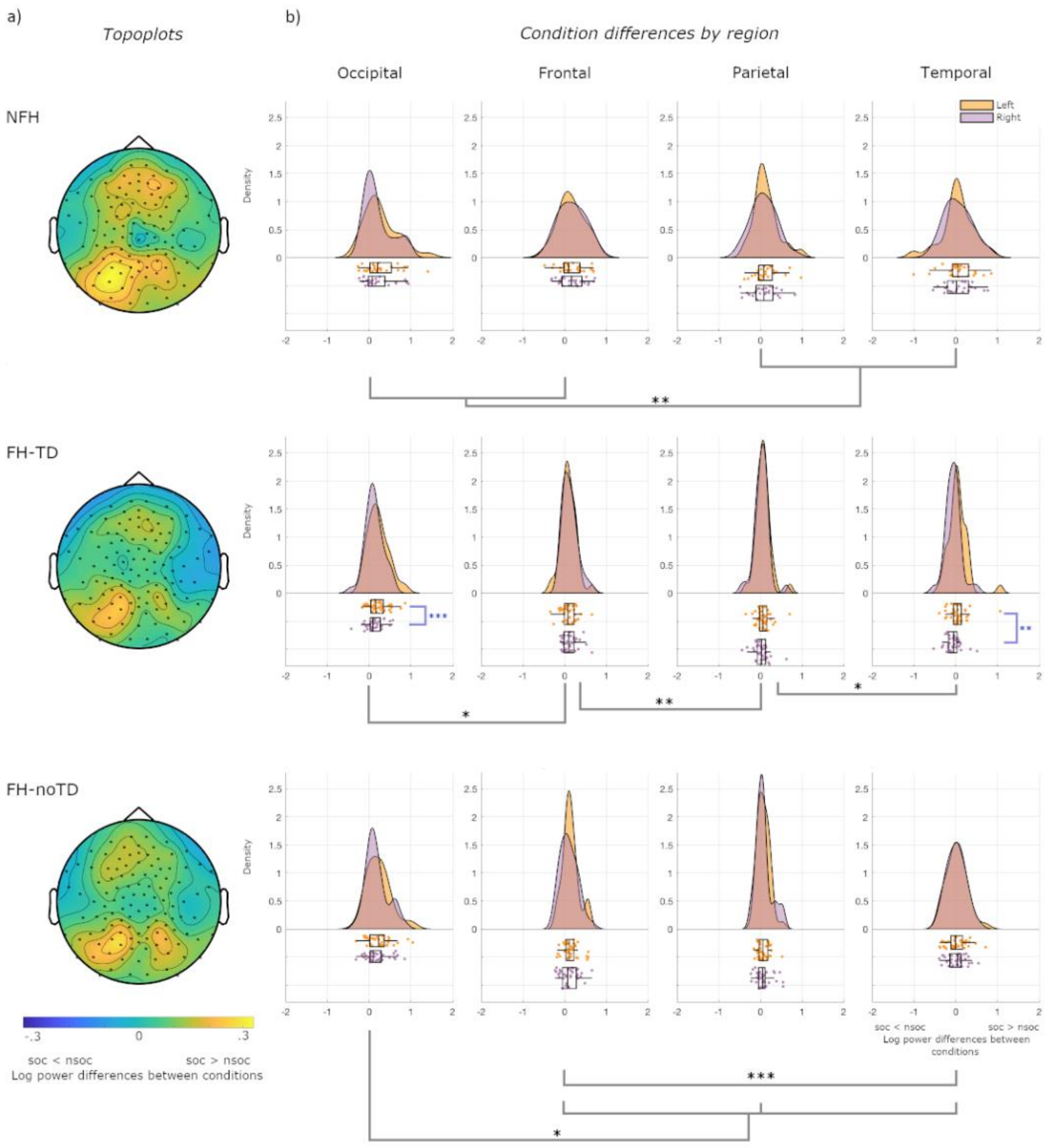

Figure SI2.2. Differences between conditions for theta power
a) Topoplots displaying the theta power differences between the social and non-social conditions within the NFH, NH-TD, and FH-noTD group. b) Distribution and boxplots for condition differences for theta power by region (from left to right column: occipital, frontal, parietal, and temporal) and laterality (left in orange, right in purple) in the NFH group (top row), FH-TD group (middle row), and FH-noTD group (bottom row). Positive values reflect increased theta log power for social compared to non-social videos, negative values reflect decreased theta low power during social compared to non-social videos. Comparisons marked in black represent post-hoc comparisons between regions, whilst those marked in blue represent post-hoc comparisons between hemispheres within a region (* $p < .05$, ** p < .01, *** p < .001).

In the FH-TD group, power increases with social content varied across region ($p < .0001$, $\eta_p^2 = .365$) where differentiation between conditions showed a graded effect across all regions with strongest increases in occipital regions, then frontal and then parietal regions, and lastly, in temporal regions. Power increases were also stronger in the left than right hemisphere in this group ($p < .0001$, $\eta_p^2 = .300$). Finally, the interaction between

Region and Hemisphere in this group was significant (p = .001, η_p^2 = .145). Post-hoc paired samples t-tests in separate regions revealed theta power increased with social content in the left occipital (p < .0001), right frontal (p < .0001), right occipital (p = .001), left frontal (p = .002), and left parietal (p = .022) region, whilst power decreased with social content in the right temporal region (p = .042). Theta power did not differ between conditions in the left temporal (p = .194) and right parietal (p = .306) region.

In the FH-noTD group, only the effect of Region was significant (p < .0001, η_p^2 = .333). Increases with social content were stronger across occipital regions compared to frontal, temporal and parietal regions. Frontal regions displayed stronger increases with social content than temporal regions.

To summarise the pattern of results, both FH groups showed significantly weaker Social/Non-Social differentiation over frontal than occipital regions, whilst the NFH group showed equal differentiation over occipital and frontal regions. The FH-TD group was differentiated from the FH-noTD group by more pronounced lateralization of effects with stronger modulations in the left than the right parietal and occipital areas.

2.1.1. Control analyses for spectral power findings

It is possible that the main findings of theta power modulations have been influenced by confounding factors such as age or numbers of epochs. We repeated the ANOVA analyses for power while including other variables to examine whether significance levels and effect sizes for any of the observed effects changed with these potentially confounding factors. Separate ANOVAs were performed including the number of epochs, difference in percentage of overlap among epochs between conditions, percentage of hand epochs (SI1 1.2.6), age at EEG assessment (in days), and cognitive abilities (Early Learning Composite score of the MSEL) as covariates. We first centred each of the covariates around their mean by subtracting the mean from the individual values[5]. In the last ANOVA, we included biological sex (male, female) as between-subject factor into the model.

Table SI2.5 displays an overview of the results of the control analyses for spectral theta power. When adding sex as a factor into the ANOVA, the 3-way interaction effect between condition, region, and hemisphere did no longer reach significance. This interaction did no longer reach significance when examined in males or females only. The significance level for the 4-way interaction between condition, region, hemisphere, and

group slightly decreased and the effect size slightly increased when including sex into the model. Follow-up analyses showed that this interaction was significant in the female, but not in the male subsample.

Table SI2.5. Control analyses for theta spectral power (with outcome groups)

Confounding variable	*Main effects*			
	Condition	*Group*	*Region*	*Hemisphere*
None	p < .0001, **η_p^2 = .209**	p = .076, η_p^2 = .051	p < .0001, **η_p^2 = .691**	p = .005, **η_p^2 = .079**
Number of epochs	p < .0001, **η_p^2 = .212**	p = .137, η_p^2 = .040	p < .0001, **η_p^2 = .691**	p = .005, **η_p^2 = .079**
Difference in overlap	p < .0001, **η_p^2 = .210**	p = .077, η_p^2 = .051	p < .0001, **η_p^2 = .691**	p = .005, **η_p^2 = .079**
Percentage hand epochs	p < .0001, **η_p^2 = .210**	p = .091, η_p^2 = .048	p < .0001, **η_p^2 = .691**	p = .004, **η_p^2 = .080**
Age at EEG assessment	p < .0001, **η_p^2 = .209**	p = .078, η_p^2 = .051	p < .0001, **η_p^2 = .691**	p = .005, **η_p^2 = .079**
MSEL ELC	p < .0001, **η_p^2 = .210**	p = .076, η_p^2 = .051	p < .0001, **η_p^2 = .692**	p = .005, **η_p^2 = .078**
Sex[1]	p < .0001, **η_p^2 = .181**	p = .058, η_p^2 = .058	p < .0001, **η_p^2 = .657**	p = .028, **η_p^2 = .050**
In boys	p < .0001, **η_p^2 = .265**	p = .083, η_p^2 = .105	p < .0001, **η_p^2 = .691**	p = .561, η_p^2 = .008
In girls	p = .004, **η_p^2 = .153**	p = .542, η_p^2 = .024	p < .0001, **η_p^2 = .638**	p = .017, **η_p^2 = .109**

[1] The main effect of sexreached a trend: $F(1,95) = 2.80$, $p = .097$, $\eta_p^2 = .016$, where spectral power tended to be stronger for males than females ($M_{Male} = 2.55$, se = 0.08; $M_{Female} = 2.38$, se = 0.07).

Table SI2.5 (continued). Control analyses for theta spectral power

Confounding variable	*2-way interaction effects*		
	*Con*Grp*	*Con*Reg*	*Con*Hemi*
None	p = .502, η_p^2 = .014	p < .0001, **η_p^2 = .334**	p < .0001, **η_p^2 = .125**
Number of epochs	p = .606, η_p^2 = .010	p < .0001, **η_p^2 = .336**	p < .0001, **η_p^2 = .125**
Difference in overlap	p = .502, η_p^2 = .014	p < .0001, **η_p^2 = .339**	p < .0001, **η_p^2 = .125**
Percentage hand epochs	p = .626, η_p^2 = .010	p < .0001, **η_p^2 = .336**	p < .0001, **η_p^2 = .124**
Age at EEG assessment	p = .514, η_p^2 = .014	p < .0001, **η_p^2 = .336**	p < .0001, **η_p^2 = .126**

MSEL ELC	p = .483, η_p^2 = .015	p < .0001, **η_p^2 = .337**	p < .0001, **η_p^2 = .126**
Sex	p = .749, η_p^2 = .006	p < .0001, **η_p^2 = .302**	p = .003 **η_p^2 = .086**
In boys	p = .918, η_p^2 = .004	p < .0001, **η_p^2 = .333**	p = .029, **η_p^2 = .102**
In girls	p = .399, η_p^2 = .036	p < .0001, **η_p^2 = .278**	p = .053, η_p^2 = .073

Table SI2.5 (continued). Control analyses for theta spectral power

Confounding variable	*3- and 4-way interaction effects*		
	*Reg*Hemi*Grp*	*Con*Reg*Hemi*	*Con*Reg*Hemi*Grp*
None	p = .074, η_p^2 = .038	p = .029, **η_p^2 = .032**	p = .029, **η_p^2 = .046**
Number of epochs	p = .069, η_p^2 = .039	p = .028, **η_p^2 = .032**	p = .010, **η_p^2 = .055**
Difference in overlap	p = .074, η_p^2 = .038	p = .029, **η_p^2 = .032**	p = .030, **η_p^2 = .047**
Percentage hand epochs	p = .073, η_p^2 = .039	p = .025 **η_p^2 = .033**	p = .025, **η_p^2 = .048**
Age at EEG assessment	p = .081, η_p^2 = .038	p = .027, **η_p^2 = .033**	p = .024, **η_p^2 = .048**
MSEL ELC	p = .060, η_p^2 = .040	p = .030, **η_p^2 = .032**	p = .027, **η_p^2 = .047**
Sex	p = .190, η_p^2 = .030	p = .159, η_p^2 = .018	p = .013, **η_p^2 = .055**
In boys	p = .429, η_p^2 = .043	p = .305, η_p^2 = .026	p = .934, η_p^2 = .026
In girls	p = .078, η_p^2 = .072	p = .125, η_p^2 = .039	p = .006, **η_p^2 = .113**

Overall patterns of the results remained the same when including the other variables. This suggests that theta power modulations by condition are minimally influenced by technical factors such as numbers of epochs, overlap between epochs, or composition of the non-social condition (percentage epochs from the hand condition), or by demographic factors such as age of assessment and developmental levels. Gender appears relate to the pattern of spectral power modulations in different groups, but current sample sizes and small changes in significance values and effect sizes do not allow for strong conclusions.

2.2. Connectivity modulations by social context

Global theta connectivity was increased during the social condition versus the non-social condition (p < .0001, η_p^2 = .128, Figure SI2.3a). There was no main effect of Group or interaction effect between Condition and Group for global connectivity (p's ≥ .359, and η_p^{2}'s ≤ .021).

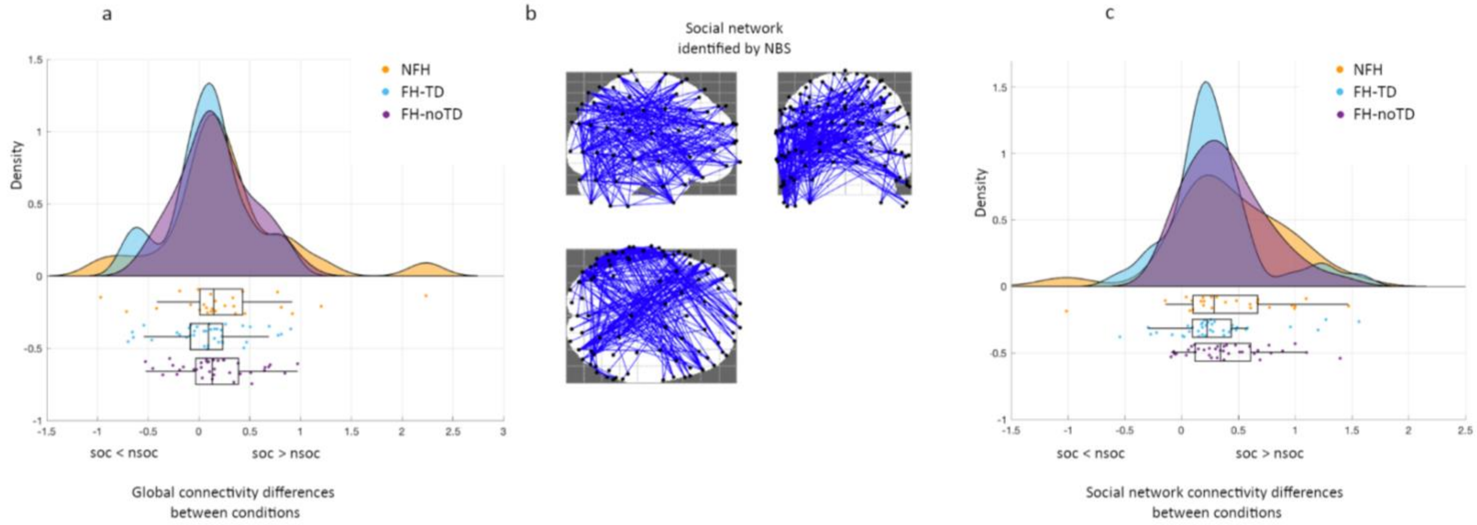

Figure SI2.3. Differences between conditions for theta connectivity
a) Distribution and boxplots for differences in global theta connectivity between the social and non-social conditions for individual infants in the NFH, FH-TD and FH-noTD group. Positive values reflect increased global theta connectivity for social compared to non-social videos, negative values reflect decreased global theta connectivity during social compared to non-social videos. b) Network of edges and nodes showing elevated connectivity during the social versus non-social condition (from the main manuscript); c) Distribution and boxplots for differences in social network connectivity between the social and non-social conditions for individual infants in the NFH, FH-TD and FH-noTD group. Positive values reflect increased theta connectivity within the interaction mask (shown in b) for social compared to non-social videos, negative values reflect decreased theta connectivity within the interaction mask during social compared to non-social videos.

Next, we compared the social network connectivity (i.e., average connectivity within the network showing elevated connectivity during social videos from the main manuscript) between the 3 groups using a 2x3 mixed ANOVA (Condition x Group). Connectivity within this network was higher for the social versus the non-social condition (p < .0001, η_p^2 = .329), as would be expected. This condition modulation however did not vary with group (p = .684, η_p^2 = .008). Overall connectivity did not vary between groups (p = .553, η_p^2 = .012).

2.2.1. Control analyses for connectivity findings

Next, we investigated whether the connectivity results may have been affected by confounding factors, such as spectral power, and age, among others. We performed multiple ANOVAs on the transformed connectivity data for these analyses, consistent with the approach of the control analyses for theta spectral power in section 2.1.1. of this document. For the connectivity control analyses, we also added spectral power differences between conditions (averaged across all channels) as potential confounding variable, in order to examine whether the connectivity findings may have resulted from the theta power modulations by condition.

The results for the control analyses for *whole-head* theta connectivity are displayed in Table SI2.7. Effect sizes and significance levels for condition, group, and the interaction were minimally influenced by the difference in spectral power between conditions, number of epochs per condition, condition differences in overlap between epochs, percentage of hand epochs, age at the EEG assessment or developmental levels as measured by the MSEL ELC. The inclusion of gender as factor slightly decreased the main effect of condition and increased the main effect of group. Further follow-up analyses in boys and girls separately revealed an effect for condition and group in the boys, whereas no other effects remained significant in the boys or girls.

Table *SI2.7*. Control analyses for whole-head theta connectivity

Confounding variable	*Effects*		
	Condition	*Group*	*C*G*
None	$p < .0001$, $\boldsymbol{\eta_p^2 = .128}$	$p = .591$, $\eta_p^2 = .011$	$p = .359$, $\eta_p^2 = .021$
Difference in theta power	$p < .0001$, $\boldsymbol{\eta_p^2 = .137}$	$p = .808$, $\eta_p^2 = .004$	$p = .547$, $\eta_p^2 = .012$
Number of epochs	$p < .0001$, $\boldsymbol{\eta_p^2 = .128}$	$p = .559$, $\eta_p^2 = .012$	$p = .362$, $\eta_p^2 = .021$
Difference in overlap	$p < .0001$, $\boldsymbol{\eta_p^2 = .130}$	$p = .591$, $\eta_p^2 = .011$	$p = .353$, $\eta_p^2 = .021$
Percentage hand epochs	$p < .0001$, $\boldsymbol{\eta_p^2 = .129}$	$p = .604$, $\eta_p^2 = .010$	$p = .340$, $\eta_p^2 = .022$
Age at EEG assessment	$p < .0001$, $\boldsymbol{\eta_p^2 = .131}$	$p = .565$, $\eta_p^2 = .012$	$p = .343$, $\eta_p^2 = .022$
MSEL ELC	$p < .0001$, $\boldsymbol{\eta_p^2 = .128}$	$p = .617$, $\eta_p^2 = .010$	$p = .366$, $\eta_p^2 = .021$

Sex *	*p* = .003<br>$\boldsymbol{\eta_p^2}$ **= .088**	*p* = .293,<br>η_p^2 = .026	*p* = .611,<br>η_p^2 = .010
In boys	*p* = .003<br>$\boldsymbol{\eta_p^2}$ **= .088**	*p* = .014,<br>$\boldsymbol{\eta_p^2}$ **= .172**	*p* = .247,<br>η_p^2 = .060
In girls	*p* = .060<br>η_p^2 = .069	*p* = .677,<br>η_p^2 = .015	*p* = .225,<br>η_p^2 = .058

* The interaction effect between group and sex reached a trend: $F(1,95) = 2.79$, $p = .066$, $\eta_p^2 = .056$, with males in the NFH group showing increased whole-head theta connectivity compared to males in both EL groups ($F(2,45) = 4.66$, $p = .014$, $\eta_p^2 = .172$; $M_{NFH} = -1.47$, std = 0.44; $M_{FH\text{-}TD} = -1.80$, std = 0.19; $M_{EL\text{-}noTD} = -1.77$, std = 0.24), whereas there are no differences between groups in the females ($F(2,50) = 0.39$, $p = .677$, $\eta_p^2 = .015$; $M_{NFH} = -1.81$, std = 0.50; $M_{FH\text{-}TD} = -1.78$, std = 0.33; $M_{EL\text{-}noTD} = -1.67$, std = 0.41).

Next, we ran control analyses for theta connectivity within the *social network* (averaged dbWPLI across electrode pairs with the social network which were log transformed, see Table SI2.8) using the same variables as for the control analyses for whole-head theta connectivity. None of the analyses revealed a change in pattern of results when including a possibly confounding variable, or factor.

Table SI2.8. Control analyses for theta connectivity within the social network

Confounding variable	*Effects*		
	Condition	*Group*	*C*G*
None	*p* < .0001,<br>$\boldsymbol{\eta_p^2}$ **= .329**	*p* = .553,<br>η_p^2 = .012	*p* = .684,<br>η_p^2 = .008
Difference in theta power	*p* < .0001,<br>$\boldsymbol{\eta_p^2}$ **= .354**	*p* = .641,<br>η_p^2 = .009	*p* = .670,<br>η_p^2 = .008
Number of epochs	*p* < .0001,<br>$\boldsymbol{\eta_p^2}$ **= .329**	*p* = .575,<br>η_p^2 = .011	*p* = .694,<br>η_p^2 = .008
Difference in overlap	*p* < .0001,<br>$\boldsymbol{\eta_p^2}$ **= .335**	*p* = .553,<br>η_p^2 = .012	*p* = .677,<br>η_p^2 = .008
Percentage hand epochs	*p* < .0001,<br>$\boldsymbol{\eta_p^2}$ **= .329**	*p* = .574,<br>η_p^2 = .011	*p* = .671,<br>η_p^2 = .008
Age at EEG assessment	*p* < .0001,<br>$\boldsymbol{\eta_p^2}$ **= .332**	*p* = .582,<br>η_p^2 = .011	*p* = .737,<br>η_p^2 = .006
MSEL ELC	*p* < .0001,<br>$\boldsymbol{\eta_p^2}$ **= .328**	*p* = .579,<br>η_p^2 = .011	*p* = .672,<br>η_p^2 = .008
Gender *	*p* < .0001,<br>$\boldsymbol{\eta_p^2}$ **= .291**	*p* = .280,<br>η_p^2 = .026	*p* = .531,<br>η_p^2 = .013

* There was a 2-way interaction effect between group and sex ($F(2,95) = 3.44$, $p = .036$, $\eta_p^2 = .068$), consistent with the uneven male: female ratios found in section 1.2 in this document.

To conclude, the connectivity control analyses revealed that the theta connectivity effects found in the main analyses are not related to differences in theta spectral power, amount of data, overlap in data segments, non-social videos watched (toy or toy with hand), age at the EEG assessment, or concurrent cognitive developmental levels.

References

1. Ozonoff, S. J. *et al.* Diagnostic stability in young children at risk for autism spectrum disorder : a baby siblings research consortium study. *J. Child Psychol. Psychiatry* **56**, 988–998 (2015).
2. Lord, C., Rutter, M. & Le Couteur, A. Autism Diagnostic Interview-Revised: a revised version of a diagnostic interview for caregivers of individuals with possible pervasive developmental disorders. *J. Autism Dev. Disord.* **24**, 659–85 (1994).
3. Lord, C. *et al. Autism Diagnostic Observation Schedule Second Edition (ADOS-2) Manual (Part 1): Modules 1-4*. (Western Psychological Services., 2012).
4. Lord, C. *et al.* The Autism Diagnostic Observation Schedule – Generic: A standard measures of social and communication deficits associated with the spectrum of autism. *J. Autism Dev. Disord.* **30**, 205–223 (2000).
5. Van Breukelen, G. J. P. & Van Dijk, K. R. A. Use of covariates in randomized controlled trials. *J. Int. Neuropsychol. Soc.* **13**, 903–904 (2007).
